# Society for Cardiovascular Magnetic Resonance 2020 Case of the Week series

**DOI:** 10.1186/s12968-021-00799-0

**Published:** 2021-10-11

**Authors:** Jason N. Johnson, Jason G. Mandell, Adam Christopher, Laura J. Olivieri, Yue-Hin Loke, Michael J. Campbell, Steve Darty, Han W. Kim, Daniel E. Clark, Benjamin P. Frischhertz, Frank A. Fish, Alison L. Bailey, Michael B. Mikolaj, Sean G. Hughes, Afiachukwu Oneugbu, Jina Chung, Joseph Burdowski, Ravi Marfatia, Xiaoming Bi, Jason Craft, Rashid A. Umairi, Faiza A. Kindi, Jason L. Williams, Michael J. Campbell, Ahmed Kharabish, Manuel Gutierrez, Monika Arzanauskaite, Marousa Ntouskou, Mahi L. Ashwath, Tommy Robinson, Jeanie B. Chiang, Jonan C. Y. Lee, M. S. H. Lee, Sylvia S. M. Chen

**Affiliations:** 1 Division of Pediatric Cardiology and Pediatric Radiology, The University of Tennessee Health Science Center, Memphis, TN USA; 2 Division of Cardiology, Children’s National Hospital, Washington, DC USA; 3 Division of Pediatric Cardiology, Duke University Medical Center, Durham, NC USA; 4 Division of Cardiology, Duke University Medical Center, Durham, NC USA; 5 Division of Cardiovascular Medicine, Vanderbilt University Medical Center, Nashville, TN USA; 6 Division of Cardiovascular Medicine, University of Tennessee College of Medicine Chattanooga/Erlanger Health System, Chattanooga, TN USA; 7 Division of Cardiology, Harbor UCLA Medical Center, Torrance, CA USA; 8 Division of Cardiology, St. Francis Hospital, Roslyn, NY USA; 9 Siemens Medical Solutions, Los Angeles, CA USA; 10 Department of Radiology, The Royal Hospital, Muscat, Oman; 11 Division of Pediatric Cardiology, Nationwide Children’s Hospital, Columbus, OH USA; 12 Radiology Department, Cairo University Hospitals, Cairo, Egypt; 13 Radiology Department, Liverpool Heart and Chest Hospital, Liverpool, UK; 14 Cardiovascular Research Center-ICCC, Hospital de La Santa Creu I Sant Pau, IIB-Sant Pau, Barcelona, Spain; 15 Division of Cardiology, University of Iowa Hospitals and Clinic, Iowa City, Iowa USA; 16 Department of Radiology and Imaging, Queen Elizabeth Hospital, Hong Kong, People’s Republic of China; 17 Department of Paediatrics, Queen Elizabeth Hospital, Hong Kong, People’s Republic of China; 18 Department of Cardiology and Adult Congenital Heart Disease, The Prince Charles Hospital, Brisbane, Australia

## Abstract

The Society for Cardiovascular Magnetic Resonance (SCMR) is an international society focused on the research, education, and clinical application of cardiovascular magnetic resonance (CMR). Case of the week is a case series hosted on the SCMR website (https://www.scmr.org) that demonstrates the utility and importance of CMR in the clinical diagnosis and management of cardiovascular disease. Each case consists of the clinical presentation and a discussion of the condition and the role of CMR in diagnosis and guiding clinical management. The cases are all instructive and helpful in the approach to patient management. We present a digital archive of the 2020 Case of the Week series of 11 cases as a means of further enhancing the education of those interested in CMR and as a means of more readily identifying these cases using a PubMed or similar search engine.

**Supplementary Information:**

The online version contains supplementary material available at 10.1186/s12968-021-00799-0.

## Introduction

Once again, a huge thank you to our wonderful team of Associate Editors and Reviewers for the Society for Cardiovascular Magnetic Resonance (SCMR) website “Case of the Week” series. This year, the cases were predominantly from the United States, along with international cases from Egypt, Oman, United Kingdom, Spain and Hong Kong. There was a mixture of adult and pediatric cases, demonstrating the broad utility of cardiovascular magnetic resonance (CMR) in assessing and diagnosing cardiovascular disease. Unexpected and rare diagnoses by CMR were highlighted in 2020, thus providing important information in guiding clinical management. Cases appropriate for Case of the Week are those which demonstrate the role of CMR in context of clinical presentation, diagnosis and management; and the way it complements other imaging modalities [1]. Please continue to submit your best illustrative cases to: https://scmr.org/page/SubmitCase and enjoy the 2020 cases!

## Case 1: 4D flow CMR to identify and characterize an intracardiac shunt

### Clinical history

A 15 year old asymptomatic girl was followed by cardiology for a small perimembranous ventricular septal defect (VSD). A routine follow-up transthoracic echocardiogram (TTE) demonstrated spontaneous closure of the VSD, as well as right atrial (RA) and right ventricular (RV) enlargement. Significant volume loading from a VSD would cause left heart dilation which was not seen. The unexpected right heart dilation suggested a previously unnoticed atrial level shunt. She was referred for surgical closure of a presumed secundum atrial septal defect (ASD). However, on review of imaging prior to surgery, an enlarged coronary sinus was noted.

A pre-operative TTE confirmed the atrial shunt and dilated coronary sinus with mild dilation of the RA, RV, and pulmonary arteries (Additional file 1: Case 1 Movie 1). Additionally, the small perimembranous VSD was closed by tricuspid valve tissue. No left superior vena cava (SVC) was seen. The pulmonary venous return could not be confirmed by TTE.

### CMR findings

**Fig. 1:**
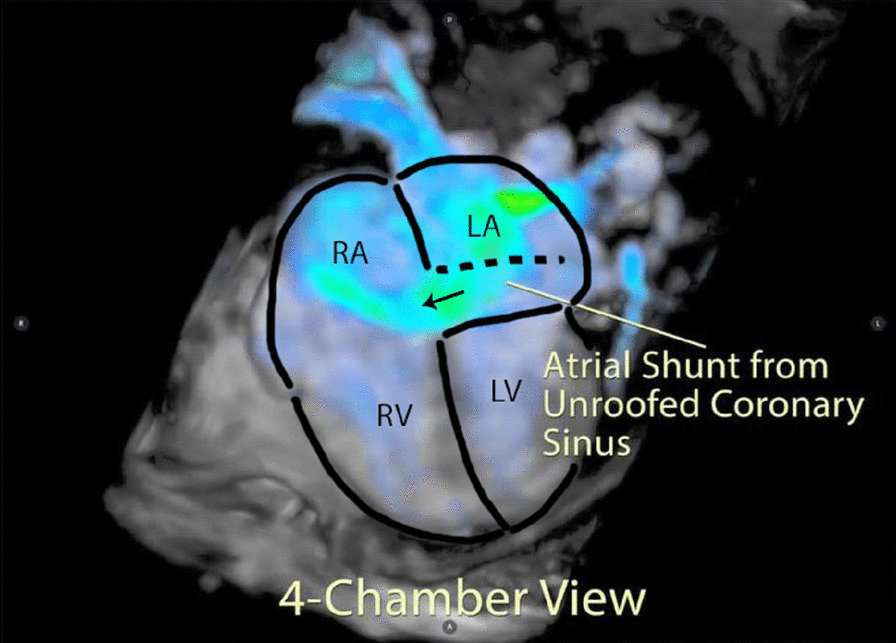
Case 1: Figure 1. 4D Flow 3D rendering. A labeled still-frame outlining the right atria (RA) and left (LA) atria, and right (RV) and left (LV) ventricles. The location of the unroofed coronary sinus is shown as a dotted line with an arrow demonstrating the direction of the shunt flow near the os of the coronary sinus

Based on the concern for a dilated coronary sinus and inadequate confirmation of normal pulmonary venous return, a CMR was performed on a 1.5 T system (Aera, Siemens Healthineers, Erlangen, Germany). Cine imaging was technically suboptimal due to respiratory and metallic artifacts, but suggested a communication from the left atrium (LA) into the proximal coronary sinus with left to right shunt into the RA, consistent with a large unroofed coronary sinus (Additional files 2, 3: Case 1 Movies 2, 3). Both the RA and RV were qualitatively enlarged. A short axis stack was not performed as the study was done to delineate the atrial shunt and venous anatomy the day prior to surgery. On an axial cine stack, the indexed RV end-diastolic volume (RVEDVI) was 160 ml/m^2^ (z-score + 5.2) which is severely enlarged. 4D flow imaging following contrast administration using Siemens WIP 785A clearly demonstrated the shunt pathway from the LA into the coronary sinus and the RA (Additional files 4, 5: Case 1 Movies 4, 5; Fig. 1: Case 1 Figure 1). There was normal pulmonary venous drainage and no left SVC. The pulmonary to systemic flow ratio was calculated to be greater than 2:1 by both 2D phase contrast and 4D flow.

### Conclusion

TTE demonstrated evidence of a left to right shunt and a dilated coronary sinus. The differential diagnosis included a coronary sinus ASD, persistence of a left SVC to the coronary sinus, and anomalous pulmonary veins to the coronary sinus. CMR cine stacks demonstrated no left SVC, normal pulmonary venous drainage, and a likely unroofed coronary sinus, suggesting the diagnosis of a coronary sinus ASD. The 4D flow imaging clearly visualized the atrial shunt and normal pulmonary venous return. The CMR findings allowed for appropriate surgical planning and counseling. The intraoperative transesophageal echocardiogram (TEE) confirmed the intracardiac anatomy (Additional file 6: Case 1 Movie 6). The surgeon noted that the coronary sinus was almost completely unroofed and repaired the defect with a pericardial patch over the mouth of the coronary sinus. The post-operative TEE demonstrated no residual shunt and normal systolic function (Additional file 7: Case 1 Movie 7). The patient recovered quickly and was discharged home on post-operative day 2.

### Perspective

ASDs are relatively common, with a reported prevalence of 1 to 2 per 1000 live births, accounting for 10 to 15% of congenital heart disease (CHD) [2]. However, coronary sinus type ASDs are much less frequent. From an anatomical standpoint, coronary sinus type ASDs are a misnomer; while their physiology is consistent with an ASD, there is no true deficiency of the atrial septum. Instead, there is a defect in the common wall between the coronary sinus and the LA which can vary from a few millimeters to complete absence of the wall of the coronary sinus. The usual method of repair is to place an autologous pericardial patch over the mouth of the coronary sinus. This results in a trivial right-to-left shunt due to coronary venous return entering the LA [3]. In cases of a coronary sinus ASD, it is common to have an associated left SVC entering the coronary sinus [4]. If the ostium of the coronary sinus is then closed, there would be a more significant right to left shunt leading to cyanosis and an alternative surgical approach is needed.

Diagnosis of coronary sinus type ASDs is challenging and often missed by TTE [5]. Additionally, a complete evaluation of systemic and venous anatomy is necessary for appropriate surgical planning and can be difficult to prove by TTE [6]. TEE can show excellent visualization of the atrial septum, though extracardiac anatomy can be difficult to see and it is a moderately invasive test. CMR can demonstrate rare intracardiac defects such as coronary sinus ASDs and quantify the hemodynamic effect of the shunt. To our knowledge, this is the first reported case of a coronary sinus ASD visualized and quantified with 4D flow CMR. 4D flow was able to clearly visualize the location of the shunt in the wall of the coronary sinus and demonstrate normal systemic and pulmonary venous drainage. 4D flow CMR has the potential to significantly increase diagnostic utility of detecting atrial shunts.

The CMR of Case 1 (Additional file CMR link, https://www.cloudcmr.com/7357-1973-4248-0193/).

## Case 2: Rare aortic arch anomaly identified by CMR in patient with Klippel–Feil syndrome

### Clinical history

A 28 year old asymptomatic male with a history of Klippel–Feil syndrome was referred for a TTE because of his history of a genetic condition. The echocardiogram revealed a dilated RA and RV and possible left-to-right atrial level shunt.

The patient was diagnosed with Klippel–Feil syndrome at 16 years of age when he was found to have multiple vertebral anomalies (fusion of C2-3, C7-T1, T2-3) as part of an evaluation for scoliosis. He subsequently had an abdominal ultrasound which revealed an absent left kidney.

### CMR findings

A CMR scan was performed on a 1.5 T (Sola, Siemens Healthineers). The CMR revealed a secundum ASD which measured 1.5 × 1.3 cm and had a Qp/Qs of 1.4 measured by phase contrast (PC) (Additional files 8, 9: Case 2 Movies 1, 2). There was moderate to severe RA enlargement and a mild to moderately dilated RV (RV end-diastolic volume 245 ml, RVEDVI 123 ml/m^2^, RV end-systolic volume 95 ml, RV end-systolic volume indexe 48 ml/m^2^) with normal systolic function (RV ejection fraction (RVEF) = 61%) (Additional file 10: Case 2 Movie 3).

**Fig. 2:**
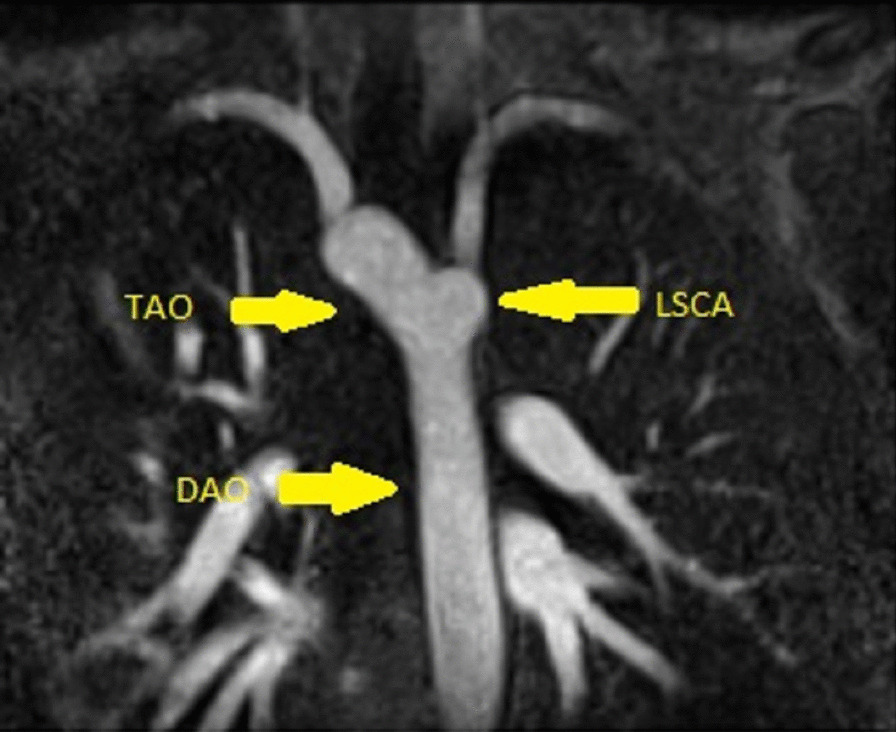
Case 2: Figure 1. Still frame angiogram in a coronal plane. Right sided transverse aortic arch (TAO) with a left descending aorta (DAo) and aberrant left subclavian artery (LSCA)

**Fig. 3:**
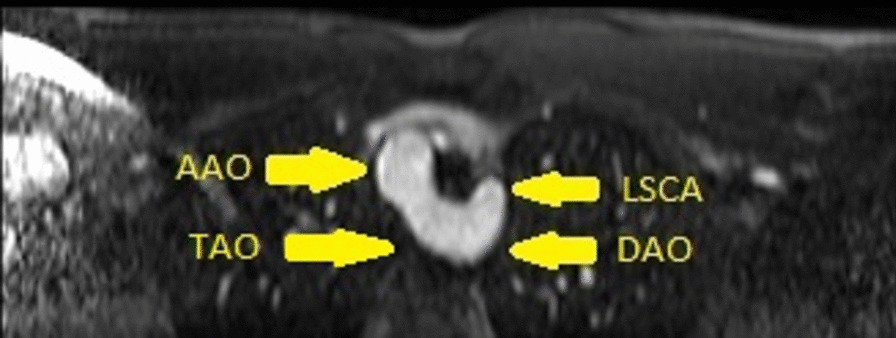
Case 2: Figure 2. Still frame angiogram in an axial plane. Right sided transverse aortic arch (TAO) with a left descending aorta (DAo) and aberrant left subclavian artery (LSCA). *AAO* ascending aorta

**Fig. 4:**
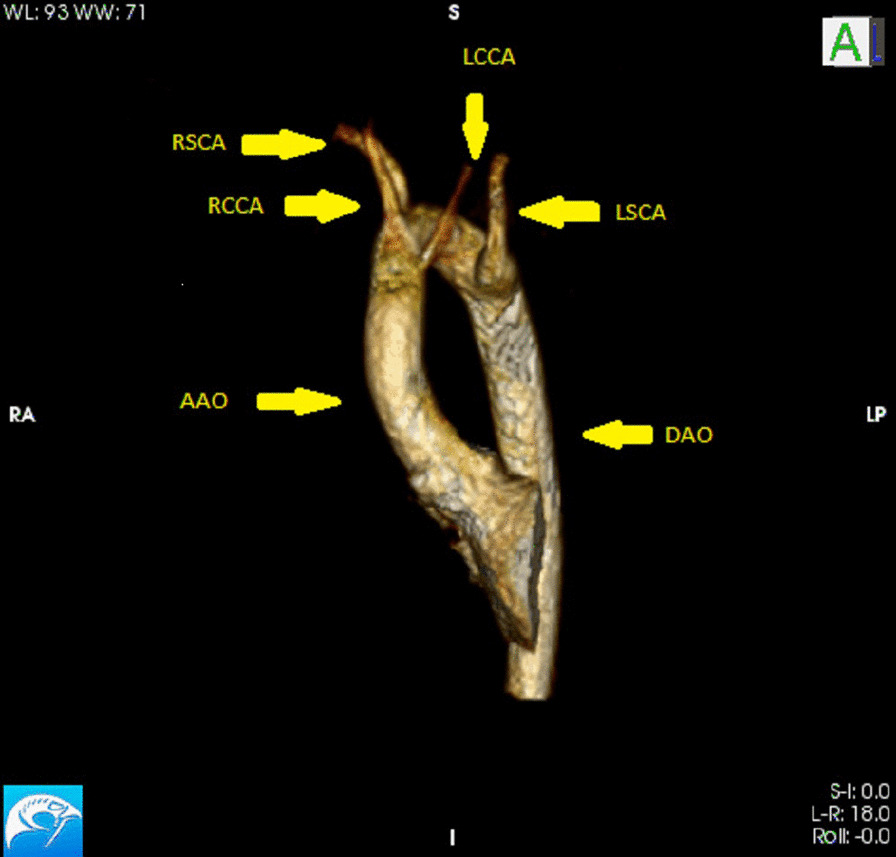
Case 2: Figure 3. Still frame 3D reconstruction of aortic arch. Right sided aortic arch with a left descending aorta (DAo) and aberrant left subclavian artery (LSCA). *AAO* ascending aorta, *LCCA* left common carotid artery, *RCCA* right common carotid artery, *RSCA* right subclavian artery

There was an incidental finding of an aortic arch abnormality. The aortic arch was right sided and took a retroesophageal course to descend on the left side of the thorax. The first head and neck vessel was the left common carotid artery followed, in order, by the right common carotid, right subclavian, and left subclavian (Additional files 11, 12 and 13: Case 2 Movies 4–6, Figs. 2, 3 and 4: Case 2 Figures 1–3).

### Conclusion

This aortic arch anatomy is consistent with a diagnosis of a right sided aortic arch with a left descending aorta and a left arterial ductus arteriosus, also known as a right aortic arch with a retroesophageal segment or circumflex retroesophageal right aortic arch [7]. There is also a secundum ASD. The patient is being evaluated for percutaneous closure of the ASD. Division of the vascular ring is not being considered because of the patient’s asymptomatic status.

### Perspective

Klippel–Feil syndrome is a musculoskeletal condition defined by the fusion of at least 2 vertebrae in the neck [8]. The syndrome is associated with a short neck, low hairline, and restricted mobility of the upper spine [8]. It can also be associated with CHD, most commonly septal defects, as in this patient [9]. Arch anomalies such as coarctation of the aorta and a right-sided aortic arch have been previously described [9, 10]. Rare reports of abnormalities of the head and neck arteries have also been described [11, 12].

This case is consistent with a right-sided aortic arch as the transverse arch crosses over the right bronchus. A right sided aortic arch with a left descending aorta is a rare aortic arch abnormality occurring in < 10% of right sided aortic arches [13]. This diagnosis is associated with other CHD in 50% of patients [13]. With this arch anatomy, the aortic arch crosses the midline at the level of the T4–5 vertebrae [7]. The first branch can be the innominate artery or the left common carotid artery with an aberrant left subclavian artery, as in this case [7]. From an embryonic standpoint, the anatomy reported here is caused by dissolution of the embryonic fourth aortic arch causing the left seventh intersegmental artery to originate from the proximal descending aorta via the left dorsal aorta [14]. Persistence of the left sixth aortic arch results in a left sided ductus arteriosus [14]. This anatomy is consistent with a vascular ring. This differential diagnosis includes a double aortic arch with an atretic left arch [7]. These two diagnosis cannot be differentiated without surgical visualization; however, this case was unlikely a double aortic arch because of the absence of a remnant of the left atretic arch from the descending aorta. CMR was critical for the diagnosis of the aortic arch abnormality in this patient.

The CMR of Case 2 (Additional file CMR link, https://www.cloudcmr.com/7657-1973-1498-0111/).

## Case 3. Ventricular tachycardia and endocardial fibroelastosis in congenital pulmonic stenosis

### Clinical history

**Fig. 5:**
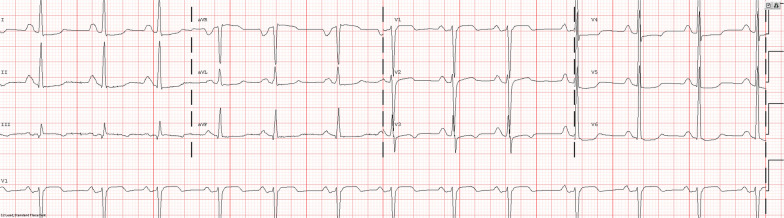
Case 3: Figure 1. 12-lead electrocardiogram (ECG). Sinus rhythm, right atrial abnormality, and diffuse nonspecific ST changes

**Fig. 6:**
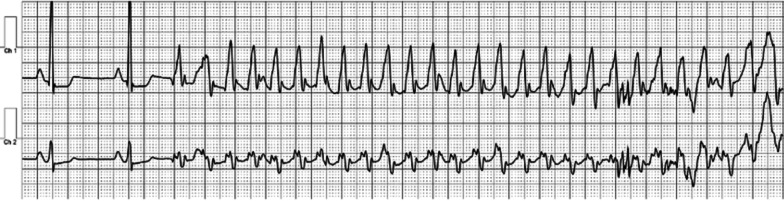
Case 3: Figure 2. Event monitor. Ventricular tachycardia at 240 beats per minute

A 24 year old male with a history of neonatal pulmonic stenosis (PS) requiring emergent valvotomy shortly after delivery presented for evaluation of palpitations and presyncope. A 12-lead electrocardiogram (ECG) revealed sinus rhythm, RA abnormality, and diffuse nonspecific ST changes (Fig. 5: Case 3 Figure 1). Event monitoring revealed 105 s of sustained ventricular tachycardia (VT) associated with presyncope (Fig. 6: Case 3 Figure 2). He was admitted to our facility for further evaluation.

TTE revealed preserved left ventricular (LV) systolic function despite abnormal septal motion, mild RV dilation, mild RV systolic dysfunction (Additional files 14, 15: Case 3 Movies 1, 2), and pulmonic regurgitation (PR) without suggestion of residual PS (peak velocity 0.6 m/s; Additional file 16: Case 3 Movie 3). PR was difficult to quantify, and diastolic septal flattening suggested RV volume overload.

### CMR findings

CMR was requested to comprehensively assess RV size/function, quantify PR, and investigate the etiology of VT. CMR revealed normal LV size (LV end-diastolic volume index 63 ml/m^2^), mild LV systolic dysfunction (LV ejection fraction (LVEF) 50%), basal septal thinning and dyskinesis, diastolic septal flattening, normal RV size (RVEDVI 102 ml/m^2^), moderate RV systolic dysfunction (RVEF 39%), normal LA size (LA volume index 26 ml/m^2^), mild tricuspid regurgitation (14 ml, 18% regurgitant fraction), and moderate PR (24 ml, 37% regurgitant fraction) (Additional files 17, 18 and 19: Case 3 Movies 4–6).

#### Scar imaging

**Fig. 7:**
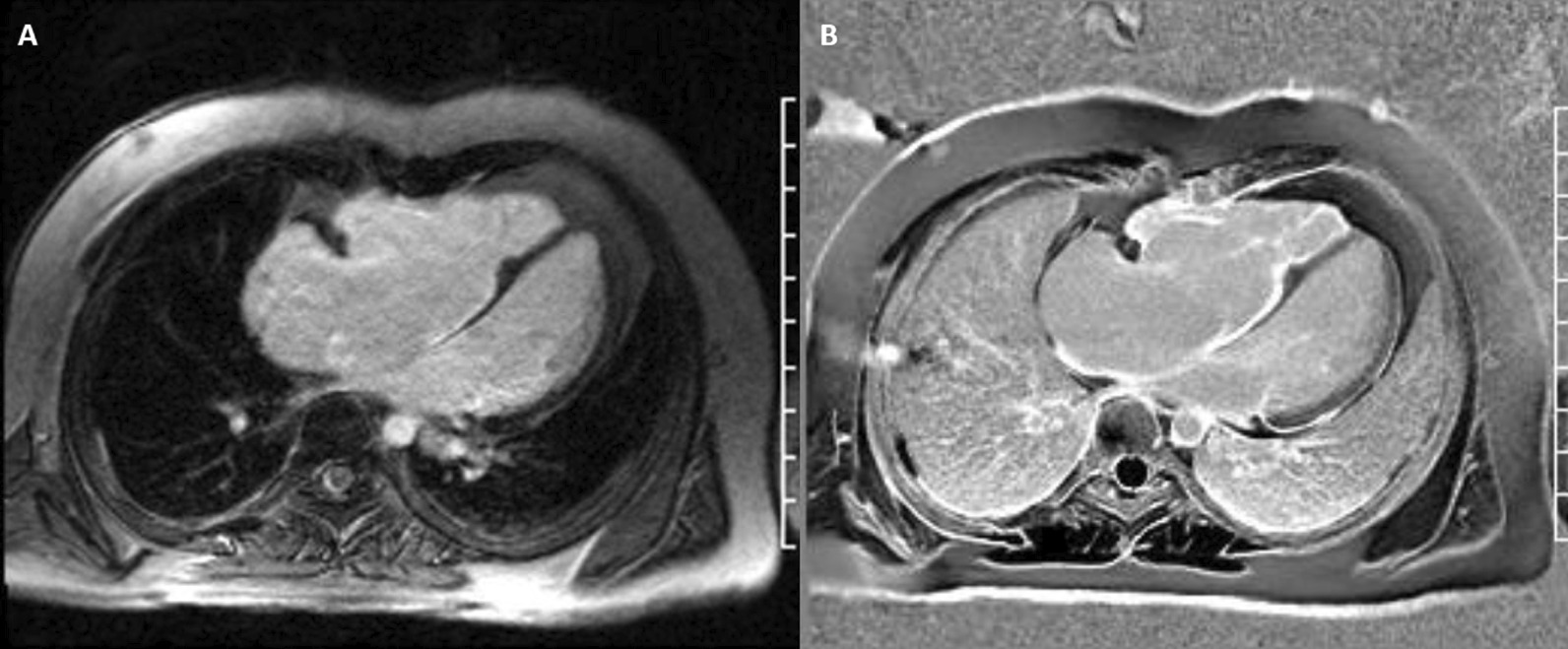
Case 3: Figure 3. CMR scar imaging. Comparison of standard “bright blood” segmented inversion recovery images (**A**) with “dark blood” technique (FIDDLE, **B**). RA and RV endocardial late gadolinium enhancement (LGE) is readily apparent on dark blood imaging

Standard “bright blood” segmented inversion recovery images were not diagnostic, but “dark blood” phase sensitive inversion recovery (PSIR) images, Flow-independent dark-blood delayed enhancement (FIDDLE), clearly showed dense RA and RV endocardial late gadolinium enhancement (LGE) (Fig. 7: Case 3 Figure 3). FIDDLE was first described by Kim and colleagues for use in detection of myocardial infarction (MI) [15]. The technique relies on selection of an inversion time (TI) with tissue magnetization more than blood, which permits simultaneous myocardial hyper-enhancement and blood-pool suppression. As seen in Fig. 7: Case 3 Figure 3, FIDDLE accentuates the contrast between enhancing myocardium and the dark blood pool, and may increase the sensitivity for detection of LGE of thin-walled structures, such as the atria, RV, and papillary apparatus.

A secondary prevention implantable cardioverter defibrillator (ICD) was planned. ICD lead deployment was complicated by unusual difficulty imbedding the fixation coil into the RV endocardium and abnormally high pacing thresholds. In conjunction with the CMR findings, a diagnosis of RV endocardial fibroelastosis (EFE) secondary to critical, congenital PS was made.

### Perspective

EFE may be either primary (idiopathic), or secondary to myocardial stress (most commonly aortic stenosis (AS) or inflammatory triggers [16]. In this patient, we suspect that EFE developed in utero secondary to critical PS and elevated RV systolic pressures. TTE is not reliable to detect EFE. Two small series of seven children have shown that CMR could detect EFE by LGE imaging [16, 17]. This case highlights the improved diagnostic utility of dark blood scar imaging for endocardial LGE assessment as compared to standard bright blood scar imaging [15, 18].

CMR may be a critical modality in the assessment of VT, especially when associated with RV dilation and/or systolic dysfunction [19, 20]. The differential diagnosis of VT in the presence of RV systolic dysfunction is broad and includes arrhythmogenic right ventricular cardiomyopathy (ARVC), sarcoidosis, MI, myocarditis, volume loading (congenital shunting, valvular regurgitation), and pressure-loading (PS, pulmonary artery hypertension) [21].

In this case, the CMR findings of dense endocardial RA and RV LGE suggested endocardial fibroelastosis, and this diagnosis was further supported by the abnormal findings during ICD implantation. CMR phase contrast velocity encoded flow imaging is superior to echocardiography in the quantification of PR, especially in patients with CHD [20]. In this particular patient, CMR revealed that abnormal septal motion was likely primarily due to basal septal thinning and fibrosis, rather than volume overload from severe PR. This case highlights the utility of dark blood LGE imaging and flow mapping in the assessment of RV size/function, morphology, and valvular insufficiency in an adult patient with repaired CHD and VT.

#### Sources of funding

Research reported in this publication was supported by the National Heart, Lung, and Blood Institute of the National Institutes of Health under Award Number T32HL007411. The content is solely the responsibility of the authors and does not necessarily represent the official views of the National Institutes of Health.

The CMR of Case 3 (Additional file CMR link, https://www.cloudcmr.com/1657-1973-0658-0142/).

## Case 4. It is not arrhythmogenic right ventricular cardiomyopathy!

### Clinical history

A 20 year old man with past medical history of spontaneous left pneumothorax presented with atypical chest pain, palpitations and lightheadedness. His TTE showed normal LVEF, with prominent RV trabeculations with mildly reduced RV function, and RV dilation (Additional file 20: Case 4 Movie 1). Subsequent bubble study done with agitated saline injection in the left antebrachial vein resulted in early opacification of a dilated coronary sinus before the RA, which was suggestive of persistent left SVC. When agitated saline was injected into the right antebrachial vein, the RA opacified before the coronary sinus confirming a normal right SVC. The RV findings raised a concern for ARVC or RV non-compaction. CMR was ordered to further evaluate the RV.

### CMR findings

A CMR and 3-dimensional time-resolved magnetic resonance angiography (MRA) showed normal LV wall thickness with mild cavity dilation and normal systolic function (LV end diastolic volume index 123.5 ml/m^2^, LVEF 61%) RV cavity size at upper limits of normal with normal systolic function (RVEDVI 103 ml/m^2^, RVEF 51%). No RV regional wall motion abnormalities or dyskinesis were present. T1, T2 weighted images pre and post contrast revealed no fatty infiltration or fibrosis of RV. Hence, CMR did not meet the criteria for ARVC or RV non-compaction. Instead, CMR and 3D MRA identified two venous anomalies: A persistent left SVC draining into the RA via the coronary sinus and the azygos continuation of the inferior vena cava (IVC) with an absent hepatic segment of IVC. (Additional files 21, 22 and 23: Case 4 Movies 2–4). Also seen is the hemiazygos vein draining into the persistent LSVC.

### Conclusion

In view of the patient’s young age and abnormal RV findings on TTE, ARVC was considered as a possible etiology of his clinical presentation. As a gold standard for RV assessment, the CMR and MRA study clarified that the patient had a normal RV size, normal RV function and tissue characteristics and also revealed two major anomalous venous connections. This case highlights the versatility of CMR and MRA in the evaluation patients with suspected RV or CHD.

### Perspective

Azygos continuation of the IVC with absence of the hepatic segment of IVC is a rare finding with a prevalence of 0.6% among patients with CHD [22]. It has been shown to be sometimes associated with other congenital anomalies like heterotaxy syndromes, cor biloculare, persistent LSVC, anomalous pulmonary venous return, double outlet RV, large ASD, and pulmonary atresia [22]. The embryonic event is theorized to be failure of the union of the right subcardinal and hepatic anastomosis, with resulting atrophy of the right subcardinal vein [22].

The renal portion of the IVC receives blood from both kidneys and passes posterior to the diaphragmatic crura to enter the thorax as the azygos vein. The azygos vein joins the SVC at the normal location in the right paratracheal space through a dilated azygos arch [23]. Since the advent of multimodality imaging this entity is now being increasingly recognized in otherwise asymptomatic patients [24]. It is important to recognize the enlarged azygos vein at the confluence with the SVC and in the retrocrural space to avoid misdiagnosis as a right-sided paratracheal mass or retrocrural adenopathy [25]. Preoperative knowledge of the anatomy may be important in planning cardiopulmonary bypass and to avoid difficulties in cardiac catheterization.

The CMR of Case 4 (Additional file CMR link, https://www.cloudcmr.com/0957-1973-4028-0165/).

## Case 5. A case of subendocardial fat deposition in a patient with eosinophilic granulomatosis with polyangitis

### Clinical history

A 57 year old female with a past medical history of eosinophilic granulomatosis with polyangiitis (EGPA), Loeffler’s endocarditis (LE), asthma, hypertension, and syncope with an implantable loop recorder presented for a follow up CMR to assess for disease progression and response to therapy. She has had known EGPA since 2007 and has had multiple prior CMR studies, which have been significant for biventricular subendocardial enhancement on LGE imaging with overlying thrombus. The patient has been chronically anticoagulated with warfarin and on chronic oral prednisone therapy since her initial diagnosis. Due to progression of myocardial eosinophilic infiltration noted on CMR in 2018, she was started on mepolizumab (Nucala) 300 mg every 4 weeks.

Upon presentation for the current study, her ECG demonstrated normal sinus rhythm and a right bundle branch block. No recent laboratory testing was available as the patient follows with hematology at a separate institution.

### CMR findings

**Fig. 8:**
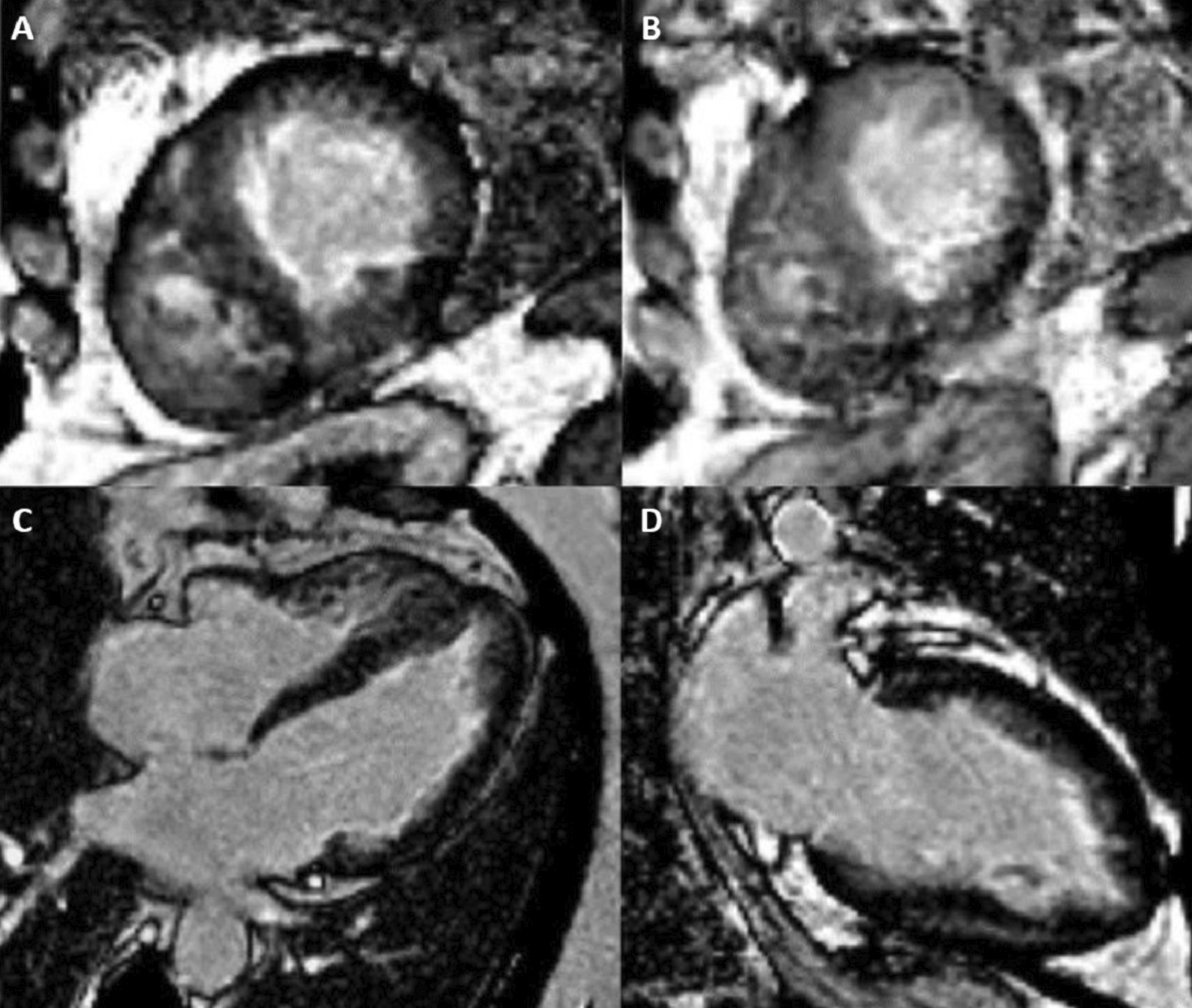
Case 5: Figure 1. Short axis mid (**A**), apical (**B**), four chamber (**C**), two chamber (**D**) LGE images acquired with GRE readout and PSIR reconstruction. Prior CMR from 2019 with subendocardial enhancement in the LV and RV apices, extending to the mid-ventricular level, and of the basal anterolateral subendocardium

CMR imaging was performed approximately one year prior in January 2019 on a 1.5 Tesla scanner (MAGNETOM Avanto, Siemens Healthineers), showing subendocardial fibrosis within the LV and RV apices, extending to the mid-ventricular level. There was also enhancement of the basal anterolateral subendocardium (Fig. 8: Case 5 Figure 1).

**Fig. 9:**
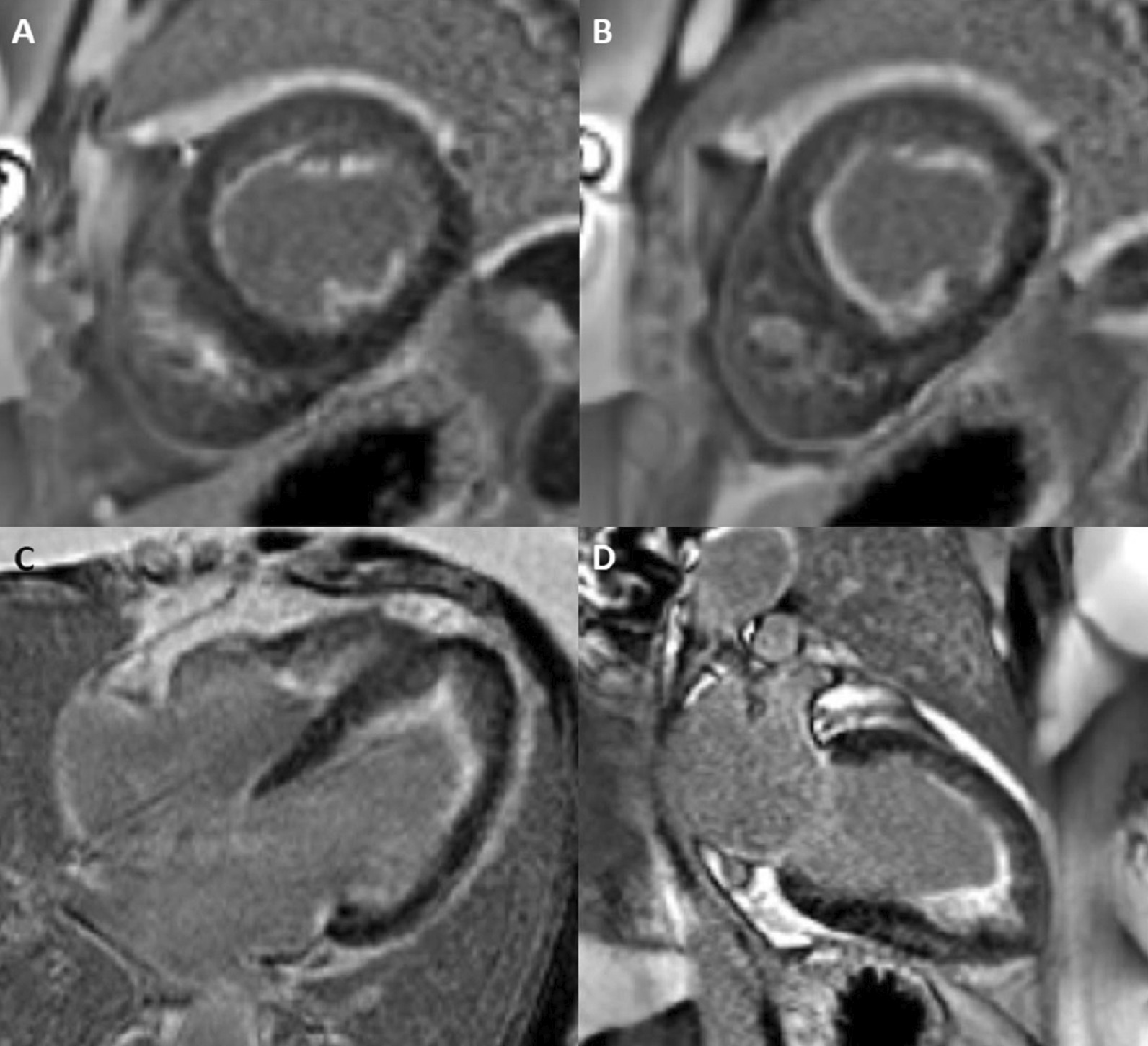
Case 5: Figure 2. Short axis mid (**A**), apical (**B**), four chamber (**C**), two chamber (**D**) LGE images acquired with gradient echo (GRE) readout and phase sensitive inversion recovery (PSIR) reconstruction. Follow up CMR from 2020 with subendocardial enhancement in the LV and RV apices. Note the improved contrast to noise between the fibrotic layer and the LV cavity blood pool when blood pool nulling is used

**Fig. 10:**
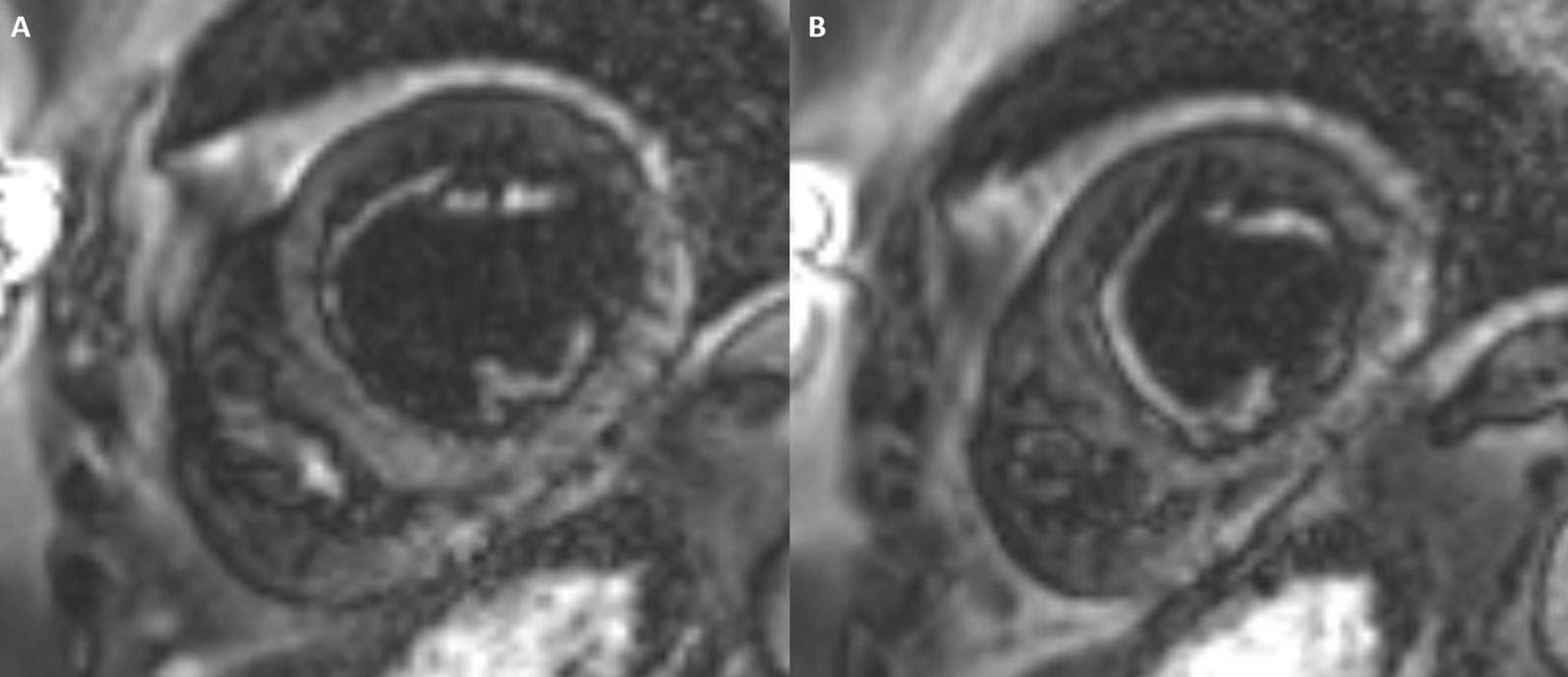
Case 5: Figure 3. Magnitude mid (**A**) and apical (**B**) short axis images corresponding to PSIR from Figure 2 Case 5. This demonstrates the blood pool nulling technique

CMR was repeated in February 2020 on a 1.5 Tesla (MAGNETOM Sola; Siemens Healthineers) for reassessment. The LVEF was 50% and the RVEF was 52%. The burden of LGE extended to the mid-ventricular level, with also involvement of the basal anterolateral subendocardium (Fig. 9: Case 5 Figure 2). LV thrombus was not identified, and this was also not present on the prior exam. Using blood pool nulling, the areas of subendocardial enhancement were more clearly demonstrated due to the increased contrast to noise ratio between fibrotic regions and the LV cavity blood pool compared to the prior exam from 2019. The magnitude images from the current study demonstrate the use of blood pool nulling (Fig. 10: Case 5 Figure 3).

**Fig. 11:**
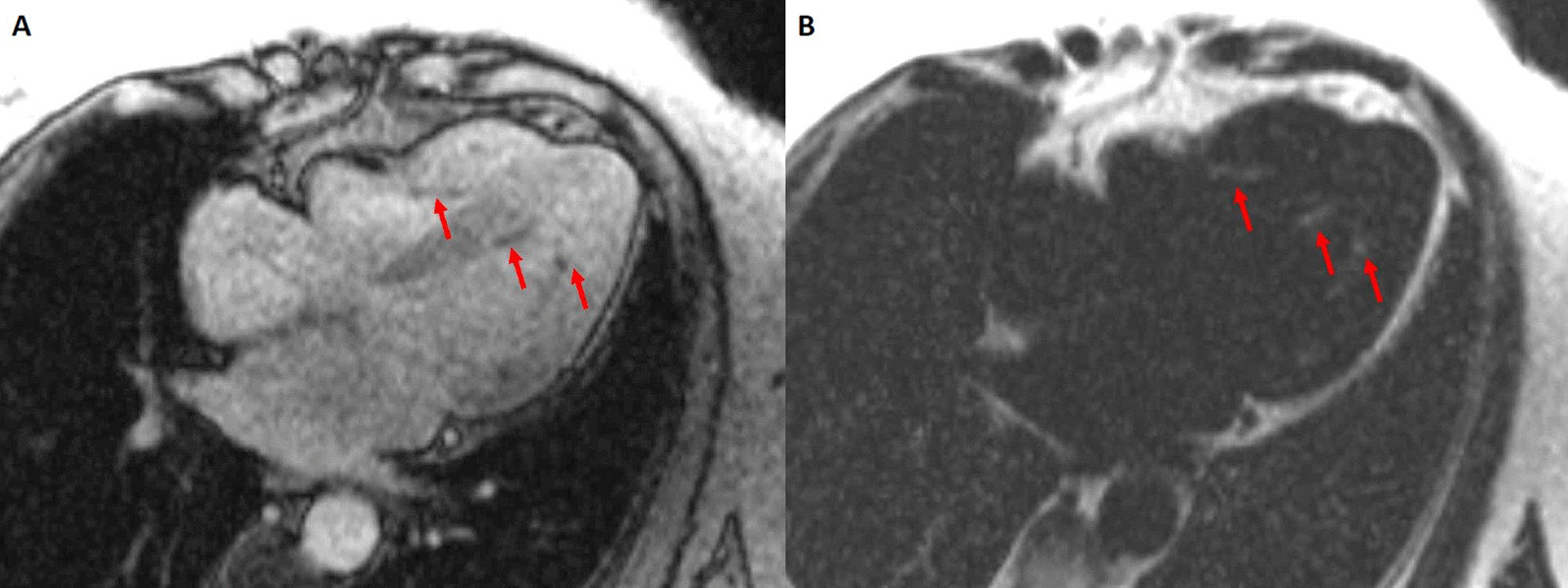
Case 5: Figure 4. Multi-echo GRE fat water separation sequence: out of phase (**A**) and fat only (**B**). Subendocardial fat deposition is depicted by the arrowheads

**Fig. 12:**
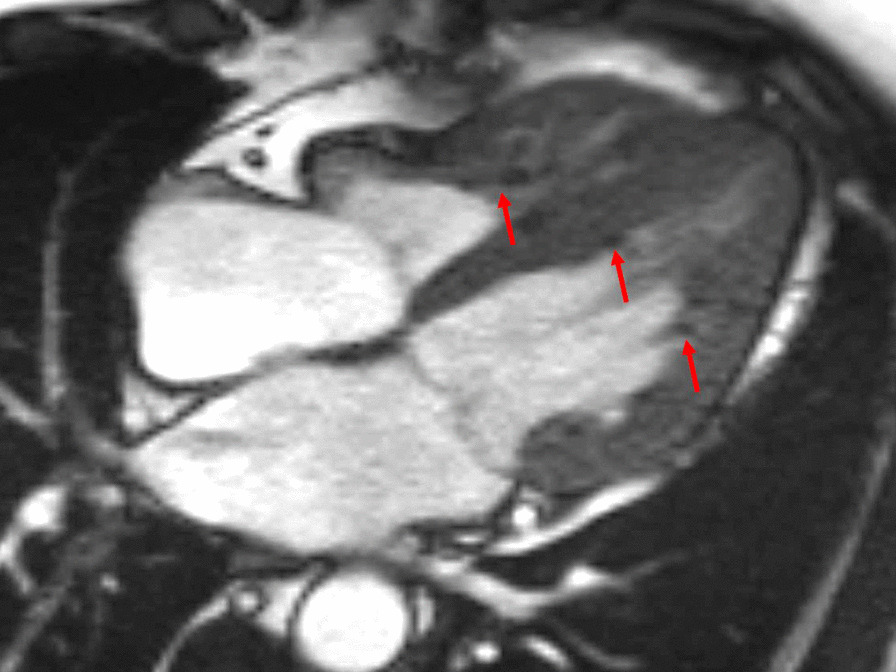
Case 5: Figure 5. Cine balanced steady state free precession (bSSFP) four chamber. Hypointense regions within the subendocardium (arrows) corresponding to scattered areas of fat deposition

A multi-echo gradient echo (GRE) fat–water separation sequence demonstrated the presence of subendocardial fat in both ventricles which can be seen in the out of phase and fat only images (Additional files 24, 25: Case 5 Movies 1, 2, Fig. 11: Case 5 Figure 4). This finding can also be appreciated on balanced steady state free precession (bSSFP) cine imaging (Additional file 26: Case 5 Movie 3, Fig. 12: Case 5 Figure 5). On first pass perfusion, there were defects at the subendocardial layer (Additional files 27, 28: Case 5 Movies 4, 5) corresponding to extensive fibrosis within the LV and RV apices.

### Conclusion

The presence of subendocardial fat, as seen in this case, has not been previously described in LE associated with EGPA. We postulate that this may represent an atypical feature of the disease or alternatively represent a finding unrelated to the disease process of LE. This finding is unlikely to represent artifact as the phenomena was demonstrated on images acquired with multiple sequence types. The case underscores the important role of CMR for tissue characterization in various disease processes. The use of multi-echo based fat–water separation techniques overcomes the limitations of spectral frequency based fat saturation. Additionally, using double/triple inversion spin echo, abnormalities at the myocardial-blood pool interface may be obscured due to incompletely suppressed blood. The Dixon technique exploits phase cycling between fat and water which completes approximately every 4.4 ms at 1.5 T. At this field strength, the resonant frequencies of fat and water are separated by 220 Hz. Therefore, at an echo time of approximately 2.2 ms, fat and water are at opposite phase (separated by ½ cycle or 180°), but are in-phase relative to one another at 4.4 ms. Fat only and water only images can be reconstructed based on this principle [26]. Finally, the use of blood pool nulling can improve the conspicuity of fibrosis at the LV blood pool/subendocardial interface to markedly improve visualization of subendocardial enhancement.

### Perspective

EGPA is a systemic small to medium vessel vasculitis, associated with asthma and peripheral eosinophilia [27]. Though primarily involving the upper respiratory tract, multi-organ involvement is often described. Cardiac manifestations can be seen in up to 62% of patients and include myocarditis, heart failure, acute coronary syndrome (ACS), pericarditis, or pericardial effusion [28]. The three stages of myocardial involvement due to hypereosinophilia include an initial necrotic phase, followed by thrombus formation and ultimately endomyocardial fibrosis.

The finding of fat deposition seen on fat water separation imaging has not been described in the literature with LE, although it has been documented as a finding in other entities that involve fibrosis of the subendocardial and mid myocardial layers (such as post-MI scar and dilated cardiomyopathy) [29, 30]. The mechanism of fatty infiltration post-MI may be due to ineffective fatty acid metabolism in injured myocytes leading to fat deposition or abnormal differentiation of cells into adipocytes in regions or fibrosis/scar [30, 31]. The potential mechanism in this case is unknown.

Blood pool nulling compared to viable myocardium nulling has been shown to improve the detection of ischemic scar. Specifically, it improves visualization of subendocardial hyperenhancement due to improved contrast to noise between the scar and LV blood pool [18, 32]. Based on this, acquisition of viable myocardium nulled images would have been reasonably expected to underestimate the subendocardial fibrosis burden, and therefore were not acquired. Patients with LE may have a dramatic response to treatment with imatinib, particularly early in the disease course [33]. Near complete reversal has been noted by CMR [34]. Contraction and/or regression of the subendocardial fibrotic layer has been postulated as an explanation. Drug therapy with Imatinib may have anti-fibrotic mechanisms acting through inhibition of the platelet derived growth factor (PDGF) receptor [35]. Serial assessment of the subendocardium by CMR as response to therapy has not been explored. PSIR blood pool nulling would provide more accurate identification of subendocardial enhancement in this context. Our patient was being followed by CMR for response to the therapy, with several treatment decisions being linked to CMR findings (for instance, LV thrombus) in the past. Overall, we have highlighted the utility of blood pool nulling and its usefulness in visualization of subendomyocardial fibrosis in our patient with LE.

The CMR of Case 5 (Additional file CMR link, https://www.cloudcmr.com/8457-1973-9078-0178/).

## Case 6. Tricuspid valve papillary fibroelastoma

### Clinical history

A 59-year-old man, admitted with a history of non-ST-elevation MI and treated with percutaneous coronary intervention (PCI), underwent TTE and found to have a mass attached to the septal leaflet of the tricuspid valve suggestive of a papillary fibroelastoma. CMR was requested to exclude other differential diagnosis such as thrombus and vegetation.

### CMR findings

**Fig. 13:**
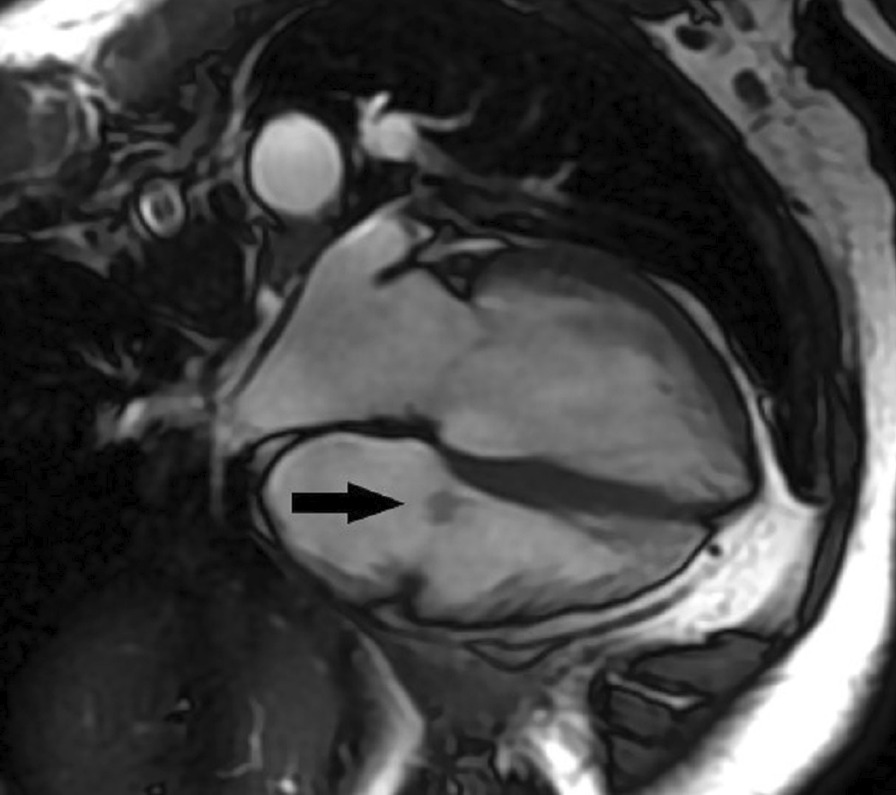
Case 6: Figure 1. Cine bSSFP four chamber. Isointense mass (arrow) on the tricuspid valve

**Fig. 14:**
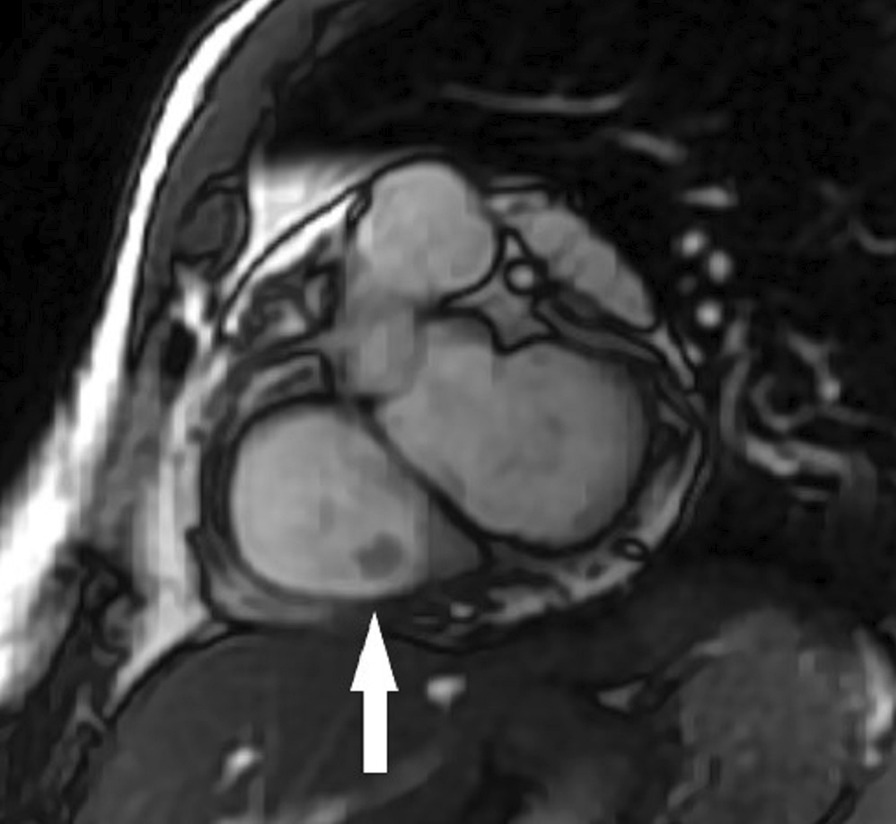
Case 6: Figure 2. Cine bSSFP short axis. Isointense mass (arrow) on the tricuspid valve

**Fig. 15:**
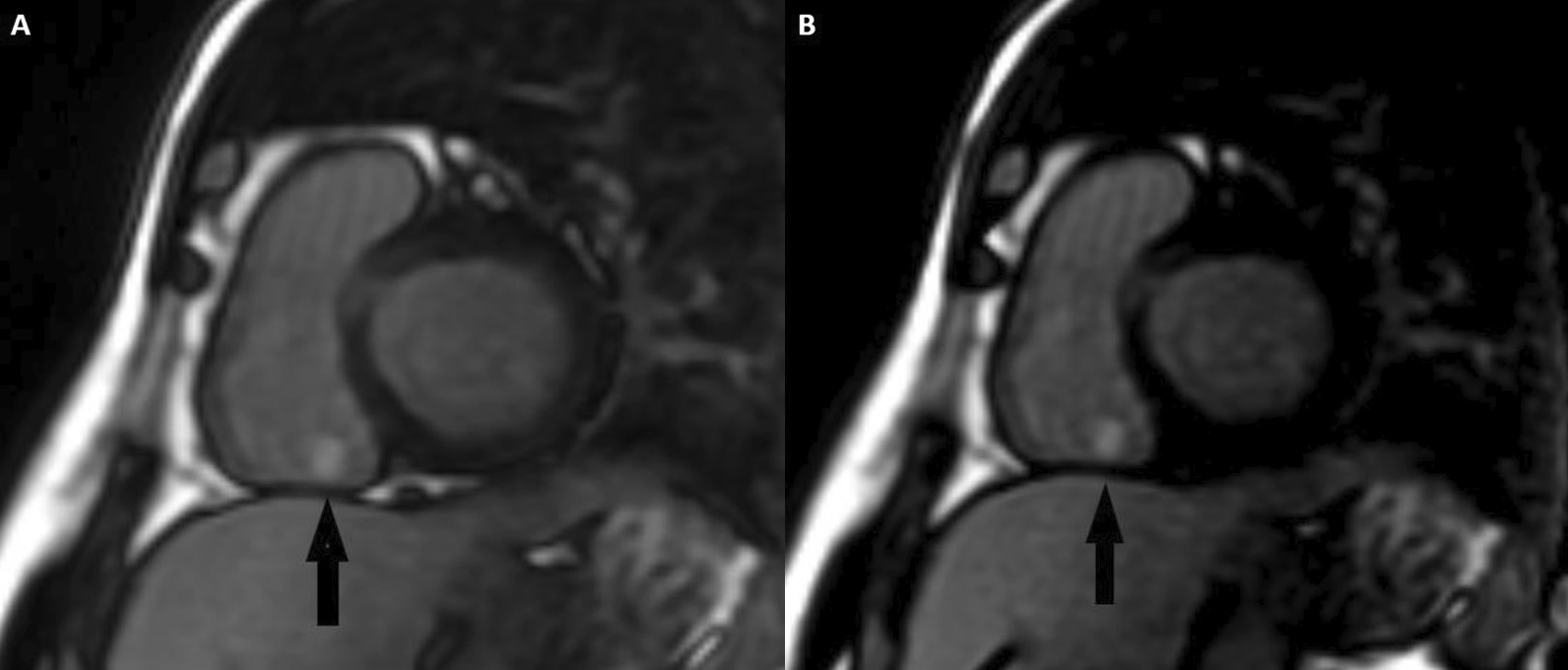
Case 6: Figure 3. LGE short axis high inversion (**A**) and null inversion (**B**) times. Hyperintense mass (arrows) on the tricuspid valve

Four chamber cine (Additional file 29: Case 6 Movie 1, Fig. 13: Case 6 Figure 1) and short axis cine (Additional file 30: Case 6 Movie 2, Fig. 14: Case 6 Figure 2) confirmed the presence of an isointense mass attached to the septal leaflet of the tricuspid valve, measuring 9 × 7 mm. LGE imaging demonstrated contrast enhancement throughout the mass (Fig. 15: Case 6 Figure 3).

### Conclusion

CMR findings of the tricuspid valve mass were suggestive of a tricuspid valve papillary fibroelastoma. The patient underwent elective surgical resection and the histopathological findings were consisting with the diagnosis of papillary fibroelastoma.

### Perspective

Cardiac tumors are rare with an estimated prevalence, according to autopsy reports, of 0.002–0.3% [36]. The majority of these tumors (75%) are benign and their clinical presentation is influenced by the size, location and embolism tendency [37]. papillary fibroelastomas are the second most common cardiac tumor and 70–80% of valvular tumors are papillary fibroelastomas [38]. Other valvular tumors include myxoma, nonbacterial thrombotic endocarditis, and infective vegetation [39].

The majority of papillary fibroelastomas are related to the aortic valve where they usually arise from the aortic side of the valve and can present with MI due to dynamic obstruction of the coronary ostia or transit ischemic attack and strokes due to thromboembolism. The mitral and tricuspid valves are the second most common sites of involvement and the tumor usually arises at the atrial side of the valves. Pulmonary valve papillary fibroelastomas are very rare with few cases reported in the literature [40].

Imaging plays an essential role in the diagnosis of cardiac papillary fibroelastomas. Most cardiac papillary fibroelastomas are detected by TTE, TEE is more sensitive compared to TTE [41]. Typically, cardiac papillary fibroelastoma appears on TTE as a rounded, oval or irregular shaped lesion with well-demarcated margin that attaches to a cardiac valve with a short mobile stalk [42]. CMR can accurately differentiate cardiac tumors from thrombus by utilizing the inversion recovery technique acquired with a prolonged inversion time (> 600 ms) after contrast administration. Tumors usually have intermediate signal intensity to the myocardium and the blood pool whereas thrombus and vegetations appear dark. On LGE sequence, thrombus and vegetations do not enhance as they are avascular structures. On the other hand, the majority of cardiac tumors are usually hyperintense owing to their vascularity [37]. Nevertheless, cardiac tumors such as lipoma, rhabdomyoma and lymphoma can show no contrast enhancement [43].

Surgical resection is usually appropriate for symptomatic patients who are candidates for surgery. However, older age, comorbid conditions and the uncertainty of the thromboembolic risk can influence the decision of surgery [41]. Long term anticoagulation is recommended for symptomatic patients who are not fit for surgery however, no data strongly validate the efficacy of this approach [41]. There are no clear guidelines for the management of papillary fibroelastomas detected incidentally. Nevertheless, many surgeons recommend surgical resection for all left-sided papillary fibroelastomas due to the potential risk of thromboembolism [44, 45]. Although extremely rare, recurrence of cardiac fibroelastoma following surgical resection has been reported [41, 46, 47].

The CMR of Case 6 (Additional file CMR link, https://www.cloudcmr.com/4557-1973-2618-0110/).

## Case 7. New murmur in a patient with pectus deformity

### Clinical history

A 14-year-old male presented to a pediatric cardiologist following referral for a new murmur heard on sports physical examination and for cardiac evaluation of connective tissue disease due to concern for Marfanoid features. His cardiac history was unremarkable, and there was no family history of connective tissue disease or sudden unexplained death. His medical history was notable for 2 years of progressive pectus carinatum. He was followed by orthopedics and was recommended for treatment with a brace. Physical exam was notable for a harsh grade III/VI systolic murmur best heard at the mid-left sternal border. Physical exam findings were also notable for a superiorly positioned pectus excavatum and an inferiorly positioned asymmetric pectus carinatum. A positive wrist sign and pes planus were noted on exam. Notably absent were hindfoot deformity, thumb sign, and reduced elbow extension. A TTE was performed, which demonstrated dynamic compression of the RV outflow tract with flow acceleration starting in the sub-valvar region and extending throughout the pulmonary trunk by Doppler assessment. The maximum velocity through the RV outflow tract was 2.0–2.5 m/s. The mitral and aortic valves were normal.

### CMR findings

**Fig. 16:**
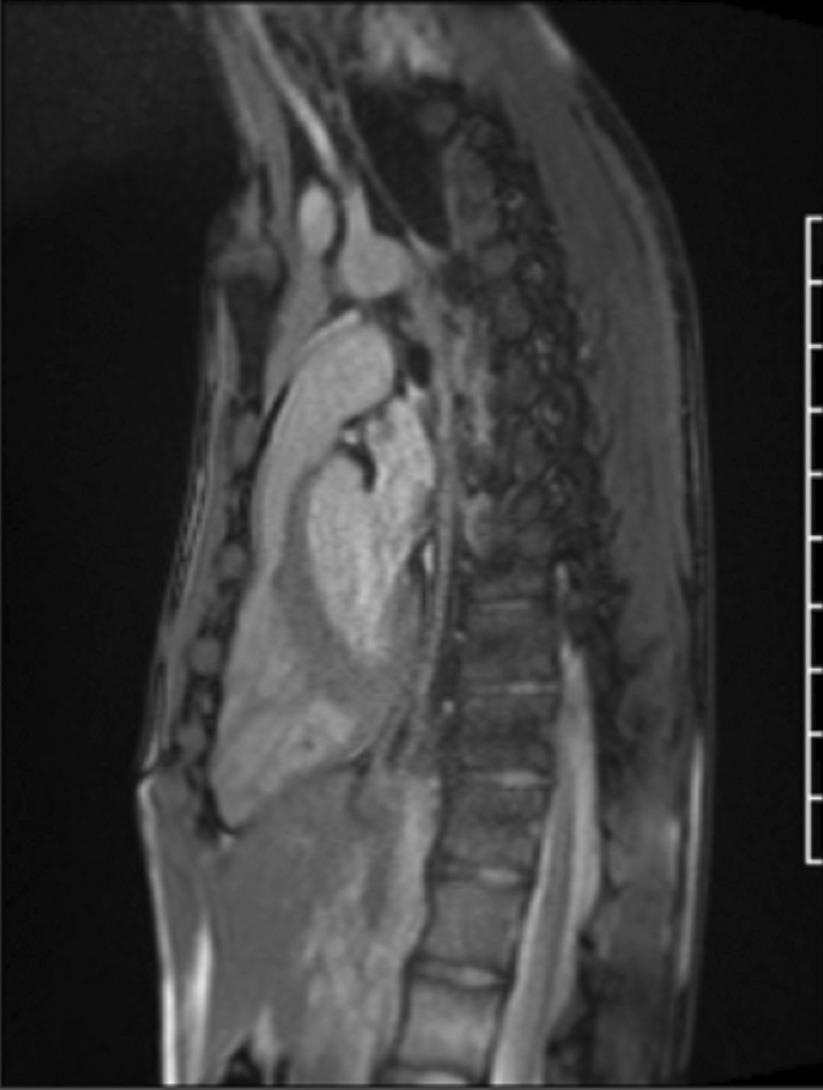
Case 7: Figure 1. Sagittal single shot bSSFP. Mild subvalvar pulmonary stenosis seen in the setting of a pectus deformity

**Fig. 17:**
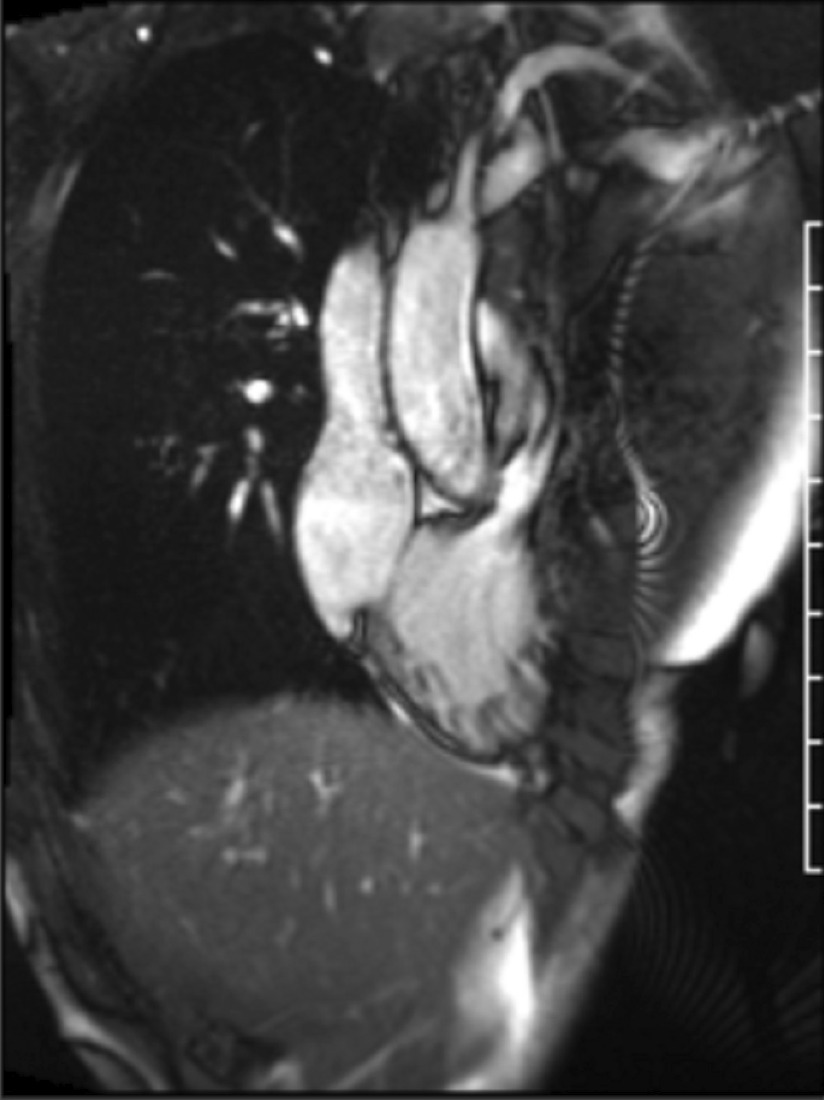
Case 7: Figure 2. RV long axis cine bSSFP at peak systole. Mild subvalvar pulmonary stenosis

A CMR scan was performed on a 3 T CMR system (Sola, Siemens Healthineers). The CMR scan revealed a prominent pectus carinatum with chest wall asymmetry (prominence of the right chest) resulting in extrinsic compression of the RV outflow tract and mild sub-pulmonary stenosis (Figs. 16, 17: Case 7 Figures 1, 2). The compression and narrowed infundibulum were demonstrated in multiple imaging planes (Additional files 31, 32: Case 7 Movies 1, 2). The peak velocity was 2.45 m/s. The anteroposterior (AP) diameter of the chest was noted to be significantly narrowed in the axial plane (Additional file 33: Case 7 Movie 3). The mitral valve and aortic valve were normal.

### Conclusion

The progression of the patient’s pectus carinatum and chest wall asymmetry during adolescence caused external compression of his RV outflow tract, creating a functionally hypoplastic infundibulum with mild sub-pulmonary stenosis. Based on his comprehensive cardiac evaluation, he did not meet clinical criteria for Marfan syndrome.

### Perspective

Pectus excavatum and pectus carinatum are common anterior chest well deformities in children [48, 49]. Pectus excavatum, the most common chest wall deformity, is defined as intrusion of the anterior chest wall into the thoracic cavity, which progresses as children age into adolescence [48, 50]. These deformities can be seen in patients with connective tissue disorders, such as the Marfan Syndrome and Noonan Syndrome [49].

CMR is a newer imaging modality used to diagnose pectus deformities and evaluate the degree of deformity in pre-surgical planning [51]. Patients can be asymptomatic; however, in one large adult cohort, 80% of patients had cardiac compression documented with symptoms of chest pain, dyspnea, palpitations, or exercise limitations [52]. Syncope has also been reported with a patient with severe pectus excavatum with compression of the RV and LA in the AP plane by CMR [53].

CMR in recent years has provided dynamic assessment of cardiac structure and function during the respiratory cycle with exaggerated septal excursion and increased LV eccentricity seen during the inspiratory phase [48, 54]. CMR also allows for a more comprehensive assessment of the effect of the pectus deformity on RV dimensions and systolic function, as ultrasound has been shown to be discrepant when evaluating ventricular function [48, 55]. Our patient was found to have normal RV function by both modalities, despite a reduced effective RV outflow tract. CMR is a useful modality to evaluate the dynamic effects of pectus deformities on cardiac anatomy and function, and should be considered in a patient with a new murmur and pectus deformity.

The CMR of Case 7 (Additional file CMR link, https://www.cloudcmr.com/8257-1973-4558-0103/).

## Case 8. Asymmetrical septal hypertrophy in cases with strong family history of hypertrophic cardiomyopathy: it is not always hypertrophic cardiomyopathy

### Clinical history

**Fig. 18:**
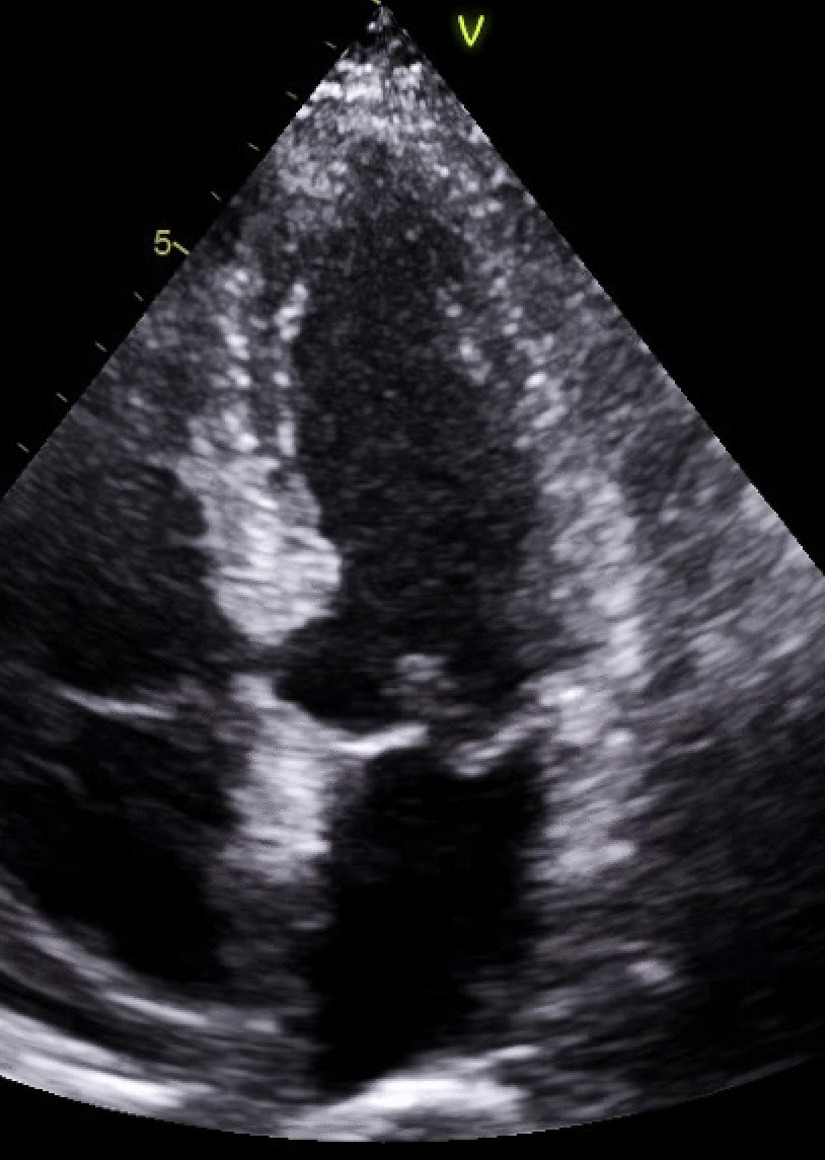
Case 8: Figure 1. Transthoracic echocardiogram (TTE) four chamber at end diastole. Mild asymmetric LV hypertrophy

We report a case of suspected hypertrophic cardiomyopathy (HCM) with a rare diagnosis that was uncovered using CMR. A 75-year-old man presented with a family history positive for HCM. His TTE revealed asymmetrical myocardial thickening (Fig. 18: Case 8 Figure 1) without outflow obstruction or papillary muscles hypertrophy, mild to moderate mitral regurgitation, and normal sized RV and LV. CMR was requested for further assessment of HCM, hemodynamics and tissue characterization.

### CMR findings

**Fig. 19:**
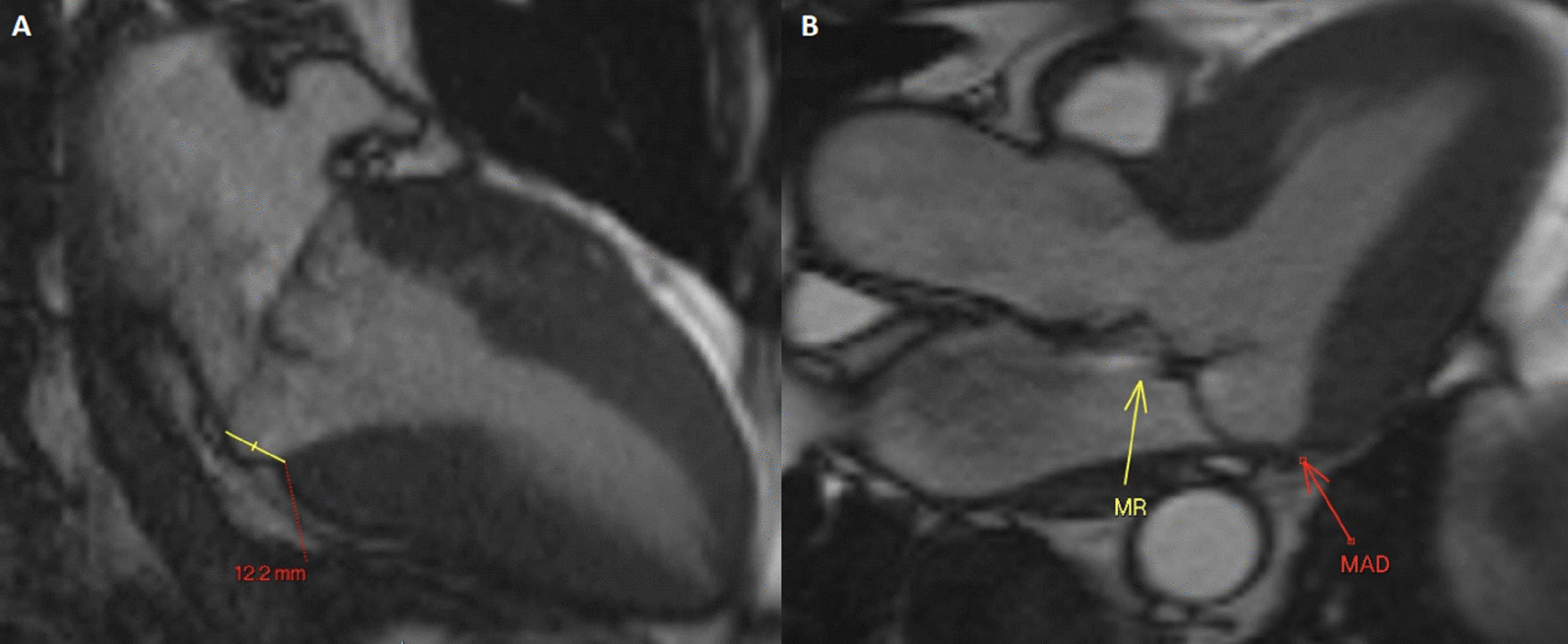
Case 8: Figure 2. Two (**A**) and three (**B**) chamber cine bSSFP at peak systole. Mitral valve prolapse (MVP) and mitral annular disjunction (MAD) are seen with a jet of mitral regurgitation (MR)

**Fig. 20:**
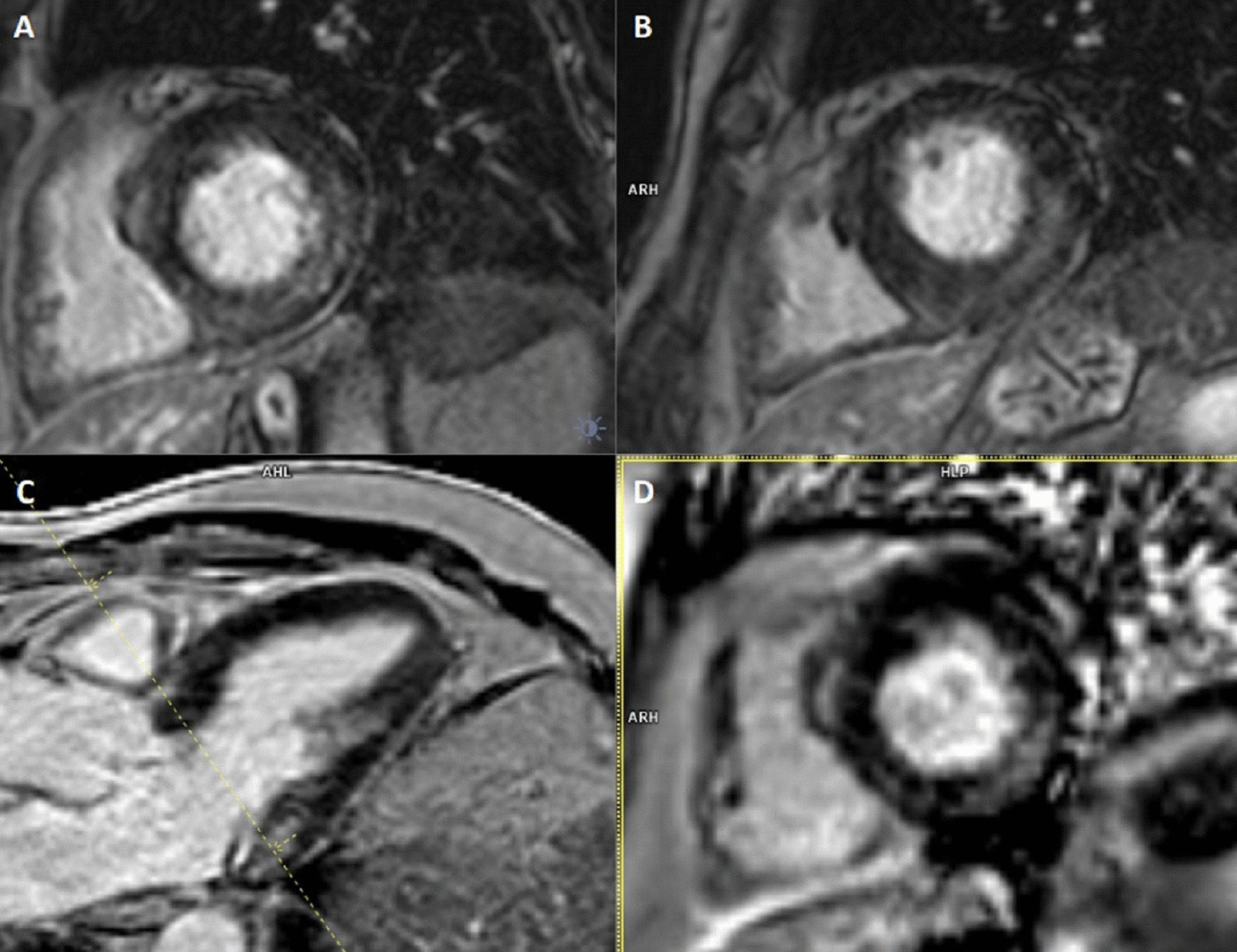
Case 8: Figure 3. LGE in short axis base (**A**, **B**), three chamber (**C**), and corresponding short axis base (**D**). Fibrosis in the inferolateral wall basal segment confirmed by the reference image through the three chamber

CMR (Fig. 19: Case 8 Figure 2, Additional files 34, 35 and 36 Case 8 Movies 1–3) showed a normal LV cavity size with mild hypertrophy of the basal septum (12 mm). The remaining myocardial segments were of normal thickness (basal anterior and anterolateral walls: 7–8 mm). There was hyperdynamic systolic function with hyperdynamic contraction of the LV basal posterolateral wall below the posterior mitral annulus. Papillary muscles were also normal. There was no obstruction in the LV outflow tract. Additionally, there was bi-leaflet mitral valve prolapse (MVP) with thickened mitral leaflets, mitral annular disjunction (MAD) and mild to moderate mitral regurgitation. Importantly, LGE showed focal annular fibrosis in the posterior mitral annulus (Fig. 20: Case 8 Figure 3).

### Conclusion

In conclusion, the pattern of ventricular hypertrophy was not typical for HCM. CMR diagnosis was MVP, MAD and focal annular myocardial fibrosis. ECG monitoring was recommended after CMR.

### Perspective

The mitral annulus is a fibro-cartilagenous structure. MVP is bowing with a displacement of > 2 mm of mitral leaflets into the LA, beyond annular plane. The pathology of mitral degeneration includes chronic activation of the valvular interstitial cells as well as the degradation of the collagen and elastin of the mitral valve by proteolytic enzymes. Histopathological types of mitral degeneration include Barlow’s disease; which is the classic type, and the fibroelastic type which leads to MAD. MAD is the separation of the atrio-mitral valve junction from the LV attachment allowing for increased mobility of the mitral valve apparatus [56]. MAD by itself is considered arrhythmogenic [57]. TTE can usually detect MVP and any associated mitral regurgitation. CMR clearly demonstrates the degree of MVP and MAD as well as the hyperdynamic basal lateral wall the basal segments’ mild focal hypertrophy [58, 59]. This asymmetric septal hypertrophy on TTE may simulate a pattern of HCM. Moreover, CMR is capable of depicting further important surrogates for instance fibrosis. The combination of MVP, MAD and focal fibrosis increases the risk of fatal heart rhythm in those patients.

The triad of MAD, MVP and focal fibrosis is arrhythmogenic, and could represent a fatal triad. It is therefore paramount to early recognize it especially in young patients using the different imaging modalities [60]. Further studies are recommended to determine whether monitoring younger patients using CMR would be essential.

The CMR of Case 8 (Additional file CMR link, https://www.cloudcmr.com/4357-1973-8568-0168/).

## Case 9. Acute chest pain with abnormal CMR

### Clinical history

A 68 year-old woman, a former smoker, presented to the emergency department after 24 h of malaise, sore throat and pleuritic chest pain. There was no other relevant past medical history. She was afebrile without other symptoms. Physical examination revealed audible crackles at bilateral lung bases with associated hypoxia.

**Fig. 21:**
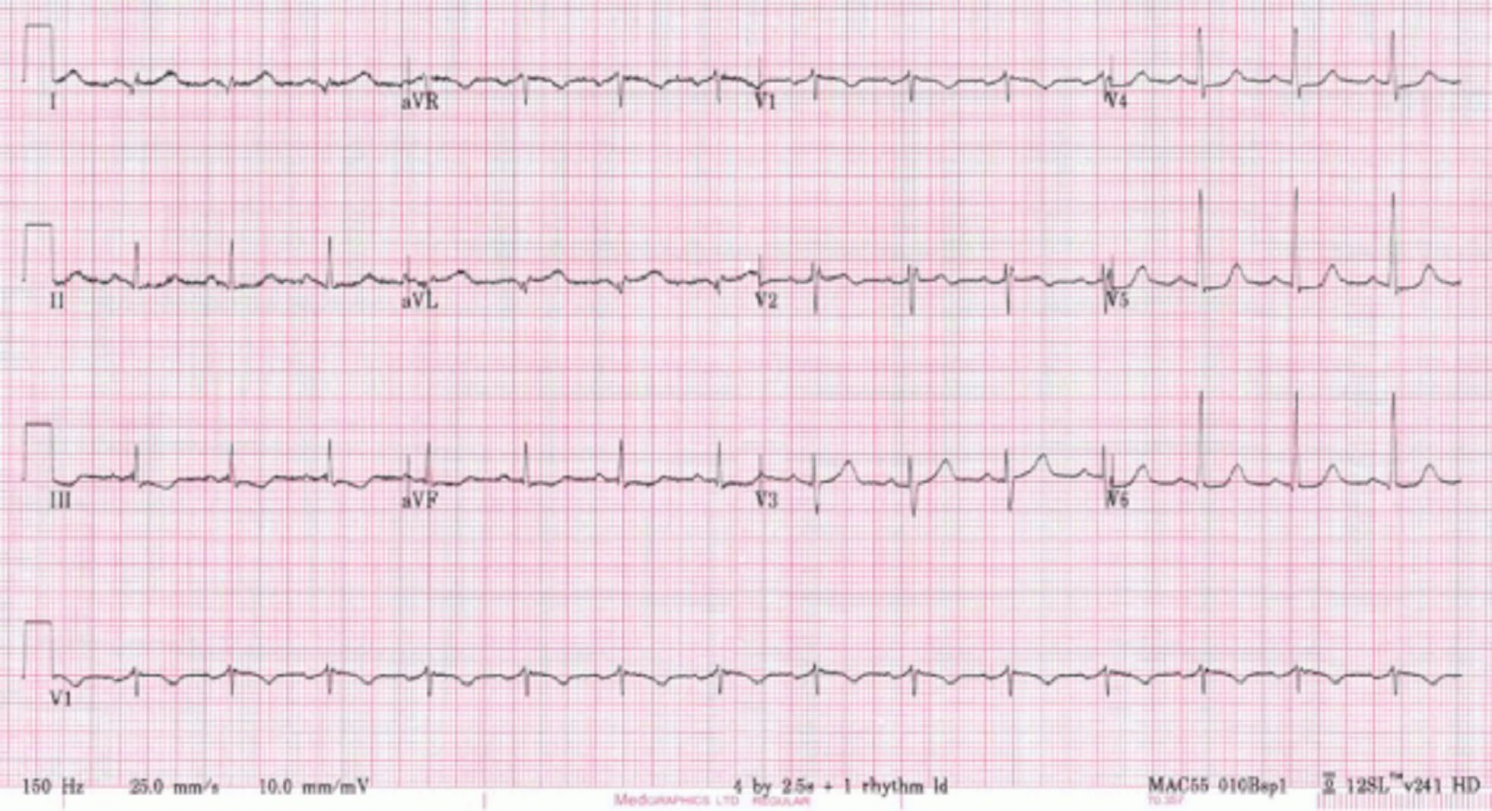
Case 9: Figure 1. Twelve lead ECG. ST depression in the lateral wall (V4–V6), T wave inversion in V1 and Q waves and ST elevation in I and aVL

ECG (Fig. 21: Case 9 Figure 1) demonstrated widespread ST depression in the inferolateral leads as well as Q waves and ST elevation in I and aVL. Portable TTE showed a severely impaired LV function with global severe hypokinesia of mid segments, hypokinesia of apical segments and a hyperdynamic base. Both cardiac troponins and creatinine kinase were severely elevated (Troponin T 1020 [< 14 ng/l]; CKMB 14 µg/l [< 5 µg/l]).

**Fig. 22:**
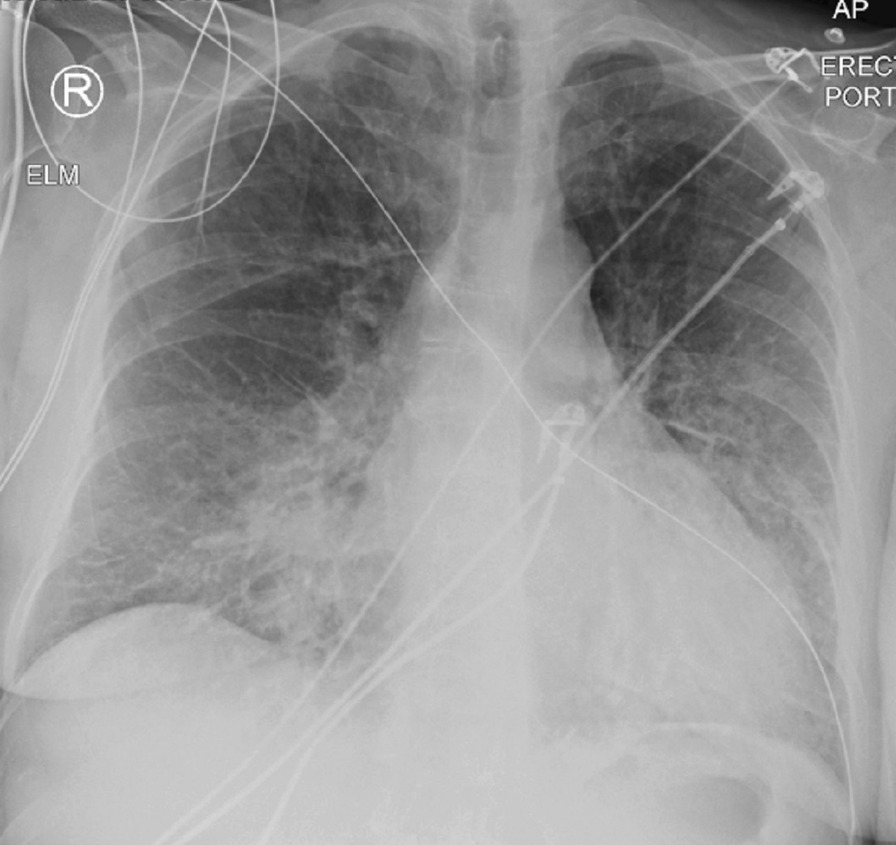
Case 9: Figure 2. Portable chest radiograph. Bilateral, peri-hilar airspace opacities and Kerley B lines consistent with acute pulmonary edema

The patient underwent an invasive angiography which did not show significant coronary artery disease (CAD). During angiography, she suffered sudden clinical deterioration with severe hypoxemia that improved after administration of high dose furosemide. A portable chest radiograph (Fig. 22: Case 9 Figure 2) on ward admission showed features consistent with pulmonary edema. Because of the absence of significant coronary disease on angiography the working diagnosis of myocardial infarction with non-obstructive coronary arteries (MINOCA) was established. Urgent CMR was requested.

### CMR findings

**Fig. 23:**
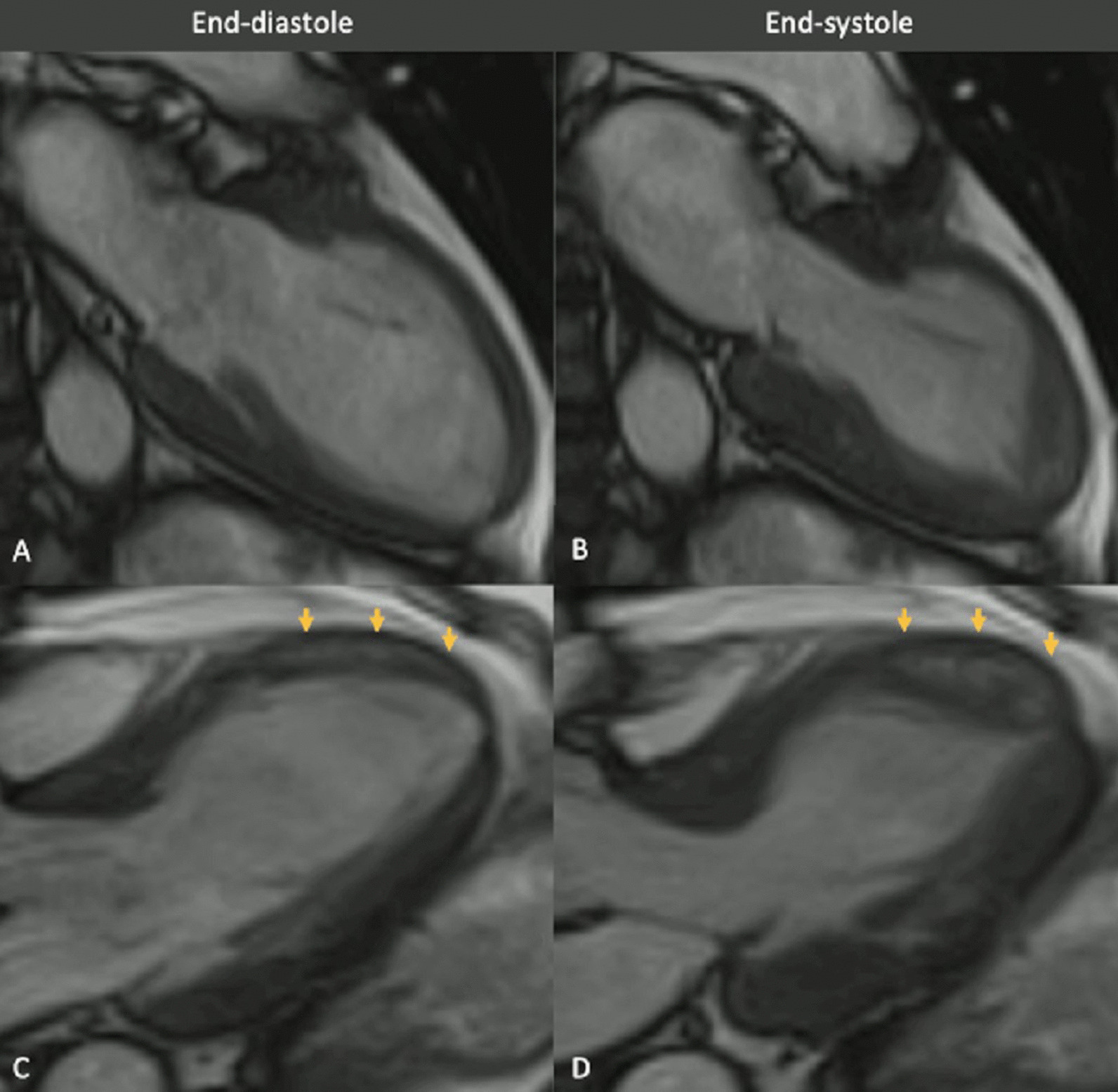
Case 9: Figure 3. Cine bSSFP two and three chamber. Two chamber (**A**, **B**) and three chamber (**C**, **D**) views of cine bSSFP in end-diastolic (left column) and end-systolic (right column). Wall motion abnormality in the mid anterior and anteroseptal walls. Note the mid to apical mid-wall myocardial hyperintensity (arrows)

Myocarditis protocol was performed at 1.5 T (Aera, Siemens Healthineers). Cine images (Fig. 23: Case 9 Figure 3) revealed severe mid-ventricular hypokinesia in keeping with the findings of the portable ultrasound on admission. The LVEF at the time of the CMR (24 h after admission) was moderately reduced (LVEF 44%).

**Fig. 24:**
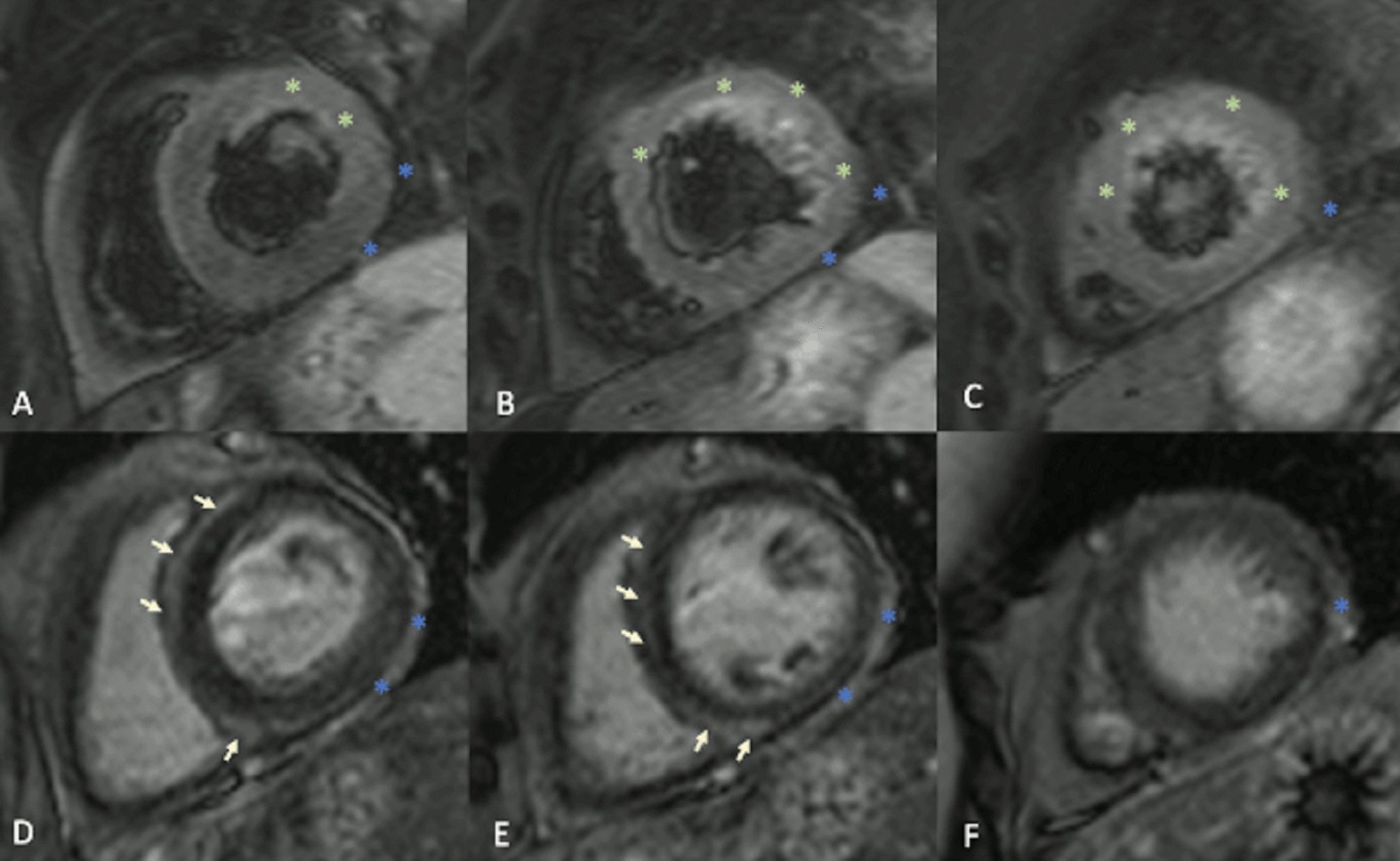
Case 9: Figure 4. T2 short tau inversion recovery (STIR) and LGE short axis. T2-STIR (**A**–**C**) and LGE (**D**–**F**) sequences in basal (left column—**A**, **D**), mid-ventricular (central column—**B**, **E**) and apical (right column—**C**, **F**) short axis views. Although relatively subtle, on STIR images there is myocardial hyperintensity (green asterisks) in the basal to apical anterior and lateral walls and mid anteroseptum, which is suggestive of myocardial edema. On LGE images there is subepicardial enhancement (arrows) in the basal to mid antero- and inferoseptum. The existence of epicardial fat (blue asterisks) might be confounded by subepicardial enhancement. Typically, epicardial fat appears hyperintense on the LGE sequence but is suppressed on STIR images

**Fig. 25:**
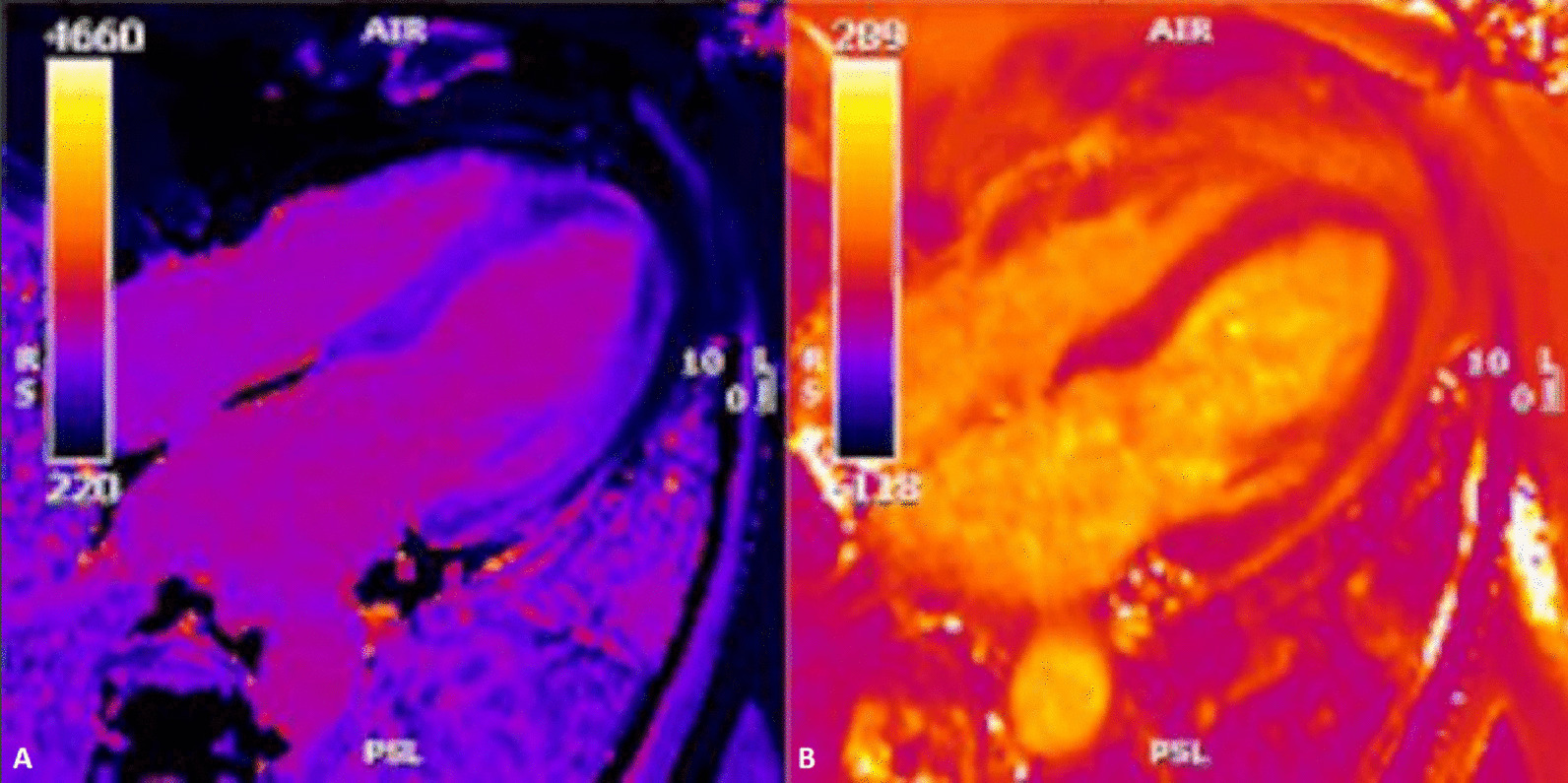
Case 9: Figure 5. Four chamber native T1 and T2 maps. On native T1 map (**A**) two areas of remarkably shortened T1 time are noted in the apical septum and apical lateral wall. As opposed to the previous tissue characterization images, the T1 time shortening is evident here. The T2 map (**B**) shows no obvious abnormalities

**Fig. 26:**
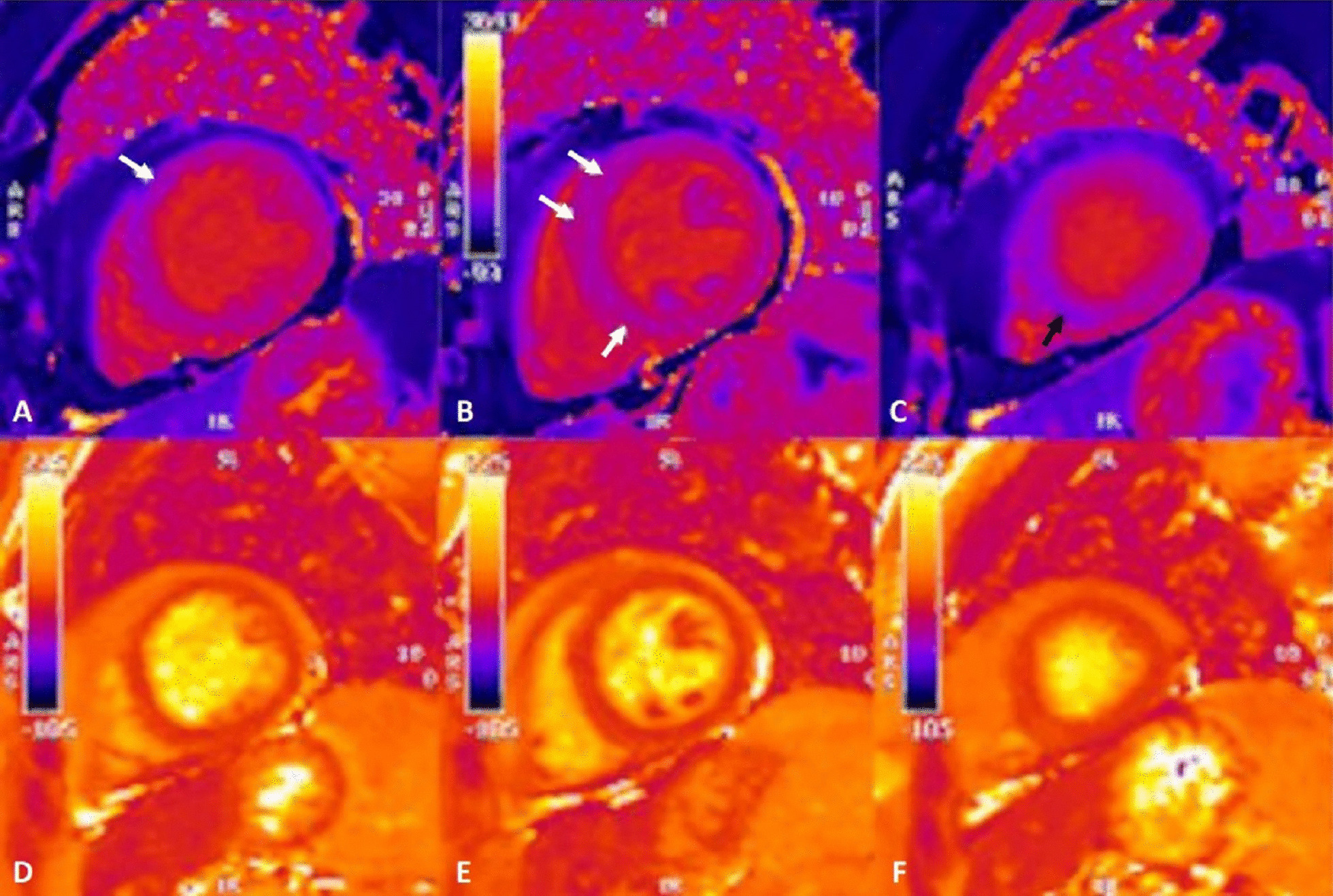
Case 9: Figure 6. Short axis native T1 and T2 maps. As with LGE sequence, in the native T1 map (**A**–**C**) there is an impression of longer times in the subepicardial basal anteroseptum and mid-wall mid-ventricular septum (white arrows). In the apical slice (**C**), there is a small area of subtle shortened T1 time in the lower part of the septum (black arrow). T2 times (**D**–**F**) appeared unremarkable in all segments

**Fig. 27:**
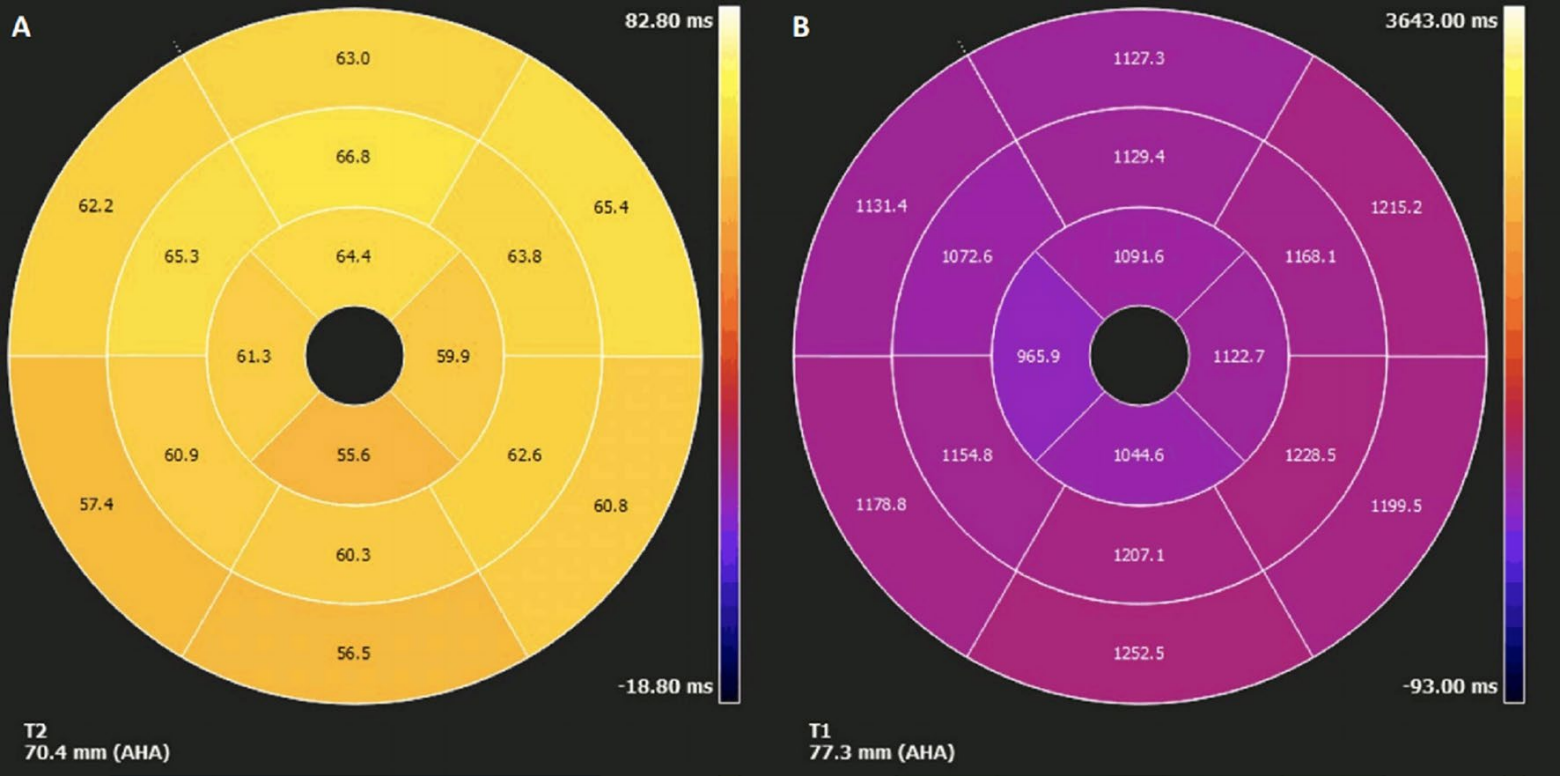
Case 9: Figure 7. Polar map of native T1 and T2 times in myocardial segments. Diffusely elevated native T1 (**A**), except where focally decreased in the apical septum. Diffusely borderline high to mildly elevated T2 times (**B**)

On T2 sequences, a mildly increased signal intensity was present predominantly in the mid anteroseptal and mid to apical anterior and lateral walls (Fig. 24: Case 9 Figure 4). Although there were no visually apparent areas of lengthened T2 times on T2 maps (Figs. 25, 26: Case 9 Figures 5, 6), a derivate segmental polar map showed diffusely borderline high to mildly elevated T2 times (55.6–66.8 ms—Fig. 27: Case 9 Figure 7). On LGE images, there was subepicardial and mid-wall enhancement in the basal to mid septal segments (Fig. 24: Case 9 Figure 4) in a non-infarct pattern. These features were most consistent with active myocardial inflammation due to myocarditis although concomitant fibrosis cannot be excluded. The clinical presentation is less consistent with stress cardiomyopathy, but this entity may also produce abnormal T1 and T2 signal on CMR due to edema and inflammation.

**Fig. 28:**
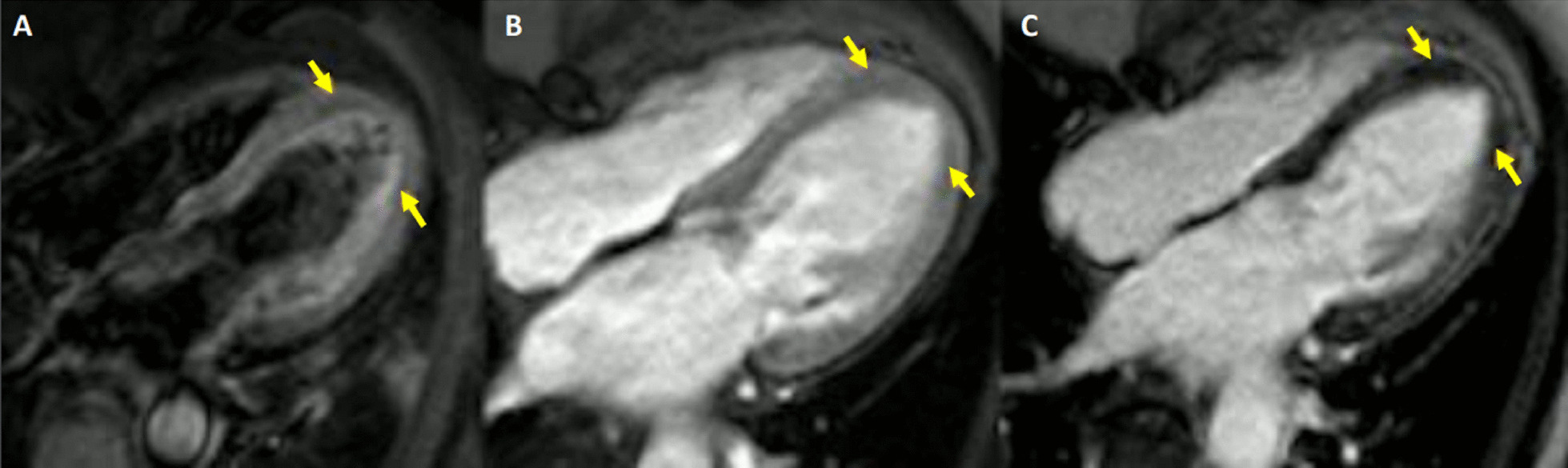
Case 9: Figure 8. Four chamber T2-STIR (**A**), early gadolinium enhancement (EGE) (**B**) and LGE (**C**) sequences. Subtle, faint areas of mid-wall apical hypointensity (yellow arrows) can be seen in all three sequences

Surprisingly, on native T1 maps (Figs. 25, 26: Case 9 Figures 5, 6) there were apical areas of shortened T1 (mean 800 ms ± 44.6 ms). Careful review of other tissue characterization sequences (T2 short tau inversion recovery (STIR), early gadolinium enhancement (EGE), LGE) allowed the identification of subtle areas of low mid-wall signal intensity matching the territories with shortened T1 times (Fig. 28: Case 9 Figure 8).

As documented in patients with acute MI, the existence of short T1 times and a hypointense core within areas of edema or LGE are suggestive of microvascular obstruction (MVO) or myocardial hemorrhage [61]. Nonetheless, in our patient there were some inconsistencies. Often, the hypointense core is easily visible and, more importantly, T2 times should be clearly abnormal. In contrast, the features in the apex (hyperintensity on bSSFP, subtle hypointense core after fat saturation and contrast administration, low T1 but normal T2 times) fit with the presence of intramyocardial fat in the context of myocardial inflammation most likely due to acute myocarditis, although stress cardiomyopathy may be possible (mild diffuse myocardial edema, native T1 increase diffusely, and areas of delayed enhancement).

### Conclusion

**Fig. 29:**
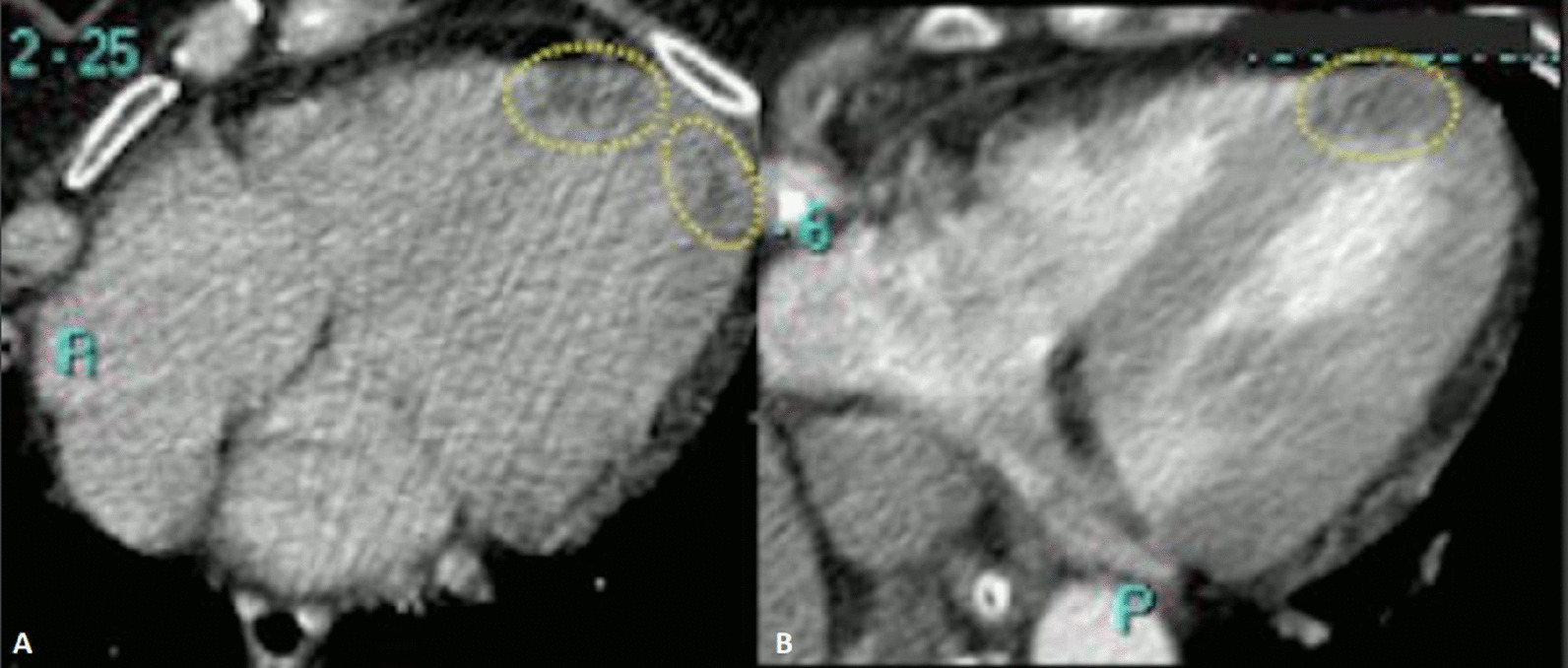
Case 9: Figure 9. Abdominal computed tomography (CT). The heart had been partially covered in two previous abdominal scans related colonic pathology (2016 (**A**) 2018 (**B**)). On both scans a focal myocardial hypodensity is visible in the same segments with abnormal signal intensity on CMR. The CT density is similar to the one showed by the pericardial and subcutaneous tissue

Given the clinical picture of atypical chest pain, LV dysfunction, ST segment changes on ECG, documented myocardial damage and consistent CMR findings (abnormal native T1, non-ischemic LGE and edema) the diagnosis of myocarditis was made. No specific etiology was identified. Stress cardiomyopathy is possible but less likely clinically. The abnormal appearances on bSSFP images and abnormal T1 times were deemed as incidental presence of myocardial fat. This was confirmed after comparing to the previous abdominal computed tomography (CT) studies where the heart had been partially covered; on these studies there were areas of focal low attenuation in the same apical segments described in the CMR study (Fig. 29: Case 9 Figure 9).

### Perspective

The diagnostic workup of acute MI without obstructed coronary arteries is not always straightforward as it encompasses multiple etiologies from overlooked coronary artery disease to non-coronary acute myocardial damage (i.e. myocarditis) [62]. CMR is the mainstay in the diagnosis of suspected myocarditis. Tissue sampling using endomyocardial biopsy is not often performed due to the invasive nature of the procedure, so it is common practice to accept the diagnosis of myocarditis when the Lake Louise criteria (revised in 2018) are met [63, 64]. These include: T1 criteria (non-ischemic pattern of LGE enhancement, prolonged T1 or increased extracellular volume) and T2 criteria (myocardial edema on STIR, lengthened T2 times). Importantly, both T1 and T2 criteria have to be present. These were identified in the case of our patient who presented with clinical, biochemical, ECG and CMR compatible findings.

However, the existence of intramyocardial fat played a confounding role initially raising the possibility of inflammation associated with severe damage. Lipomatous metaplasia of the myocardium following myocardial infarction, fibro-fatty replacement of the myocardium in ARVC, fat-containing intramyocardial lesions in tuberous sclerosis and intramyocardial lipomas are known entities of macroscopic fat tissue within the myocardium. It is also important to note that the presence of non-pathological ectopic myocardial fat is relatively common in healthy adults, and has no clinical implications [31]. Based on one cardiac CT study an incidence of up to 11% has been estimated [65]. The most usual location is the RV (free wall, RV outflow tract, moderator band or apex) however, it can also be found in the interventricular septum or LV apex. It is important to recognize this incidental fat as the features can sometimes be misleading (e.g. forming a small mass in the case of adipose degeneration of the moderator band or myocardial damage as in our case) and to correlate its presence to patient’s presentation and clinical history.

On CMR, fat can sometimes have similarities with MVO or hemorrhage, as both fat and MVO or hemorrhage cause T1 time shortening. On T2 STIR, saturated fat can give the impression of a hypointense core. Nevertheless, this appearance will be subtle whereas when the microcirculation is disrupted or there are hemoglobin products the hypointense core will generally be prominent and surrounded by obviously abnormal myocardium. The confirmation can be obtained on T2 and T2* maps: the times will be normal in cases of intramyocardial fat but abnormally low in MVO or hemorrhage. Whenever possible, previous imaging such as body CT studies will help to confirm the presence of fat, as fat has a characteristically low attenuation of − 20 to − 130 Hounsfield Units on CT, similar to the epicardial or the subcutaneous adipose tissue.

The CMR of Case 9 (Additional file CMR link, https://www.cloudcmr.com/0757-1973-9978-0149/).

## Case 10. Transient ischemic attack after left ventricular assist device explantation

### Clinical history

A 52-year old female without prior medical history was admitted with Influenza A myocarditis progressing to cardiogenic shock. She eventually required extracorporeal membrane oxygenation for biventricular failure on multiple inotropic medications. Nine days into admission, she received implantation of a HeartMate II LV assist device (LVAD) and was subsequently discharged. Over the next 6 months, myocardial function and clinical status improved. Her LVEF on TTE improved to 55–60%, with low LVAD speeds. Her LVAD was explanted.

One year later she experienced an isolated episode of transient double vision for a few minutes. A TTE did not reveal a thrombus but raised a suspicion of noncompaction and was felt to be of limited image quality. A CMR was performed to assess for non-compaction and thrombus formation.

### CMR findings

**Fig. 30:**
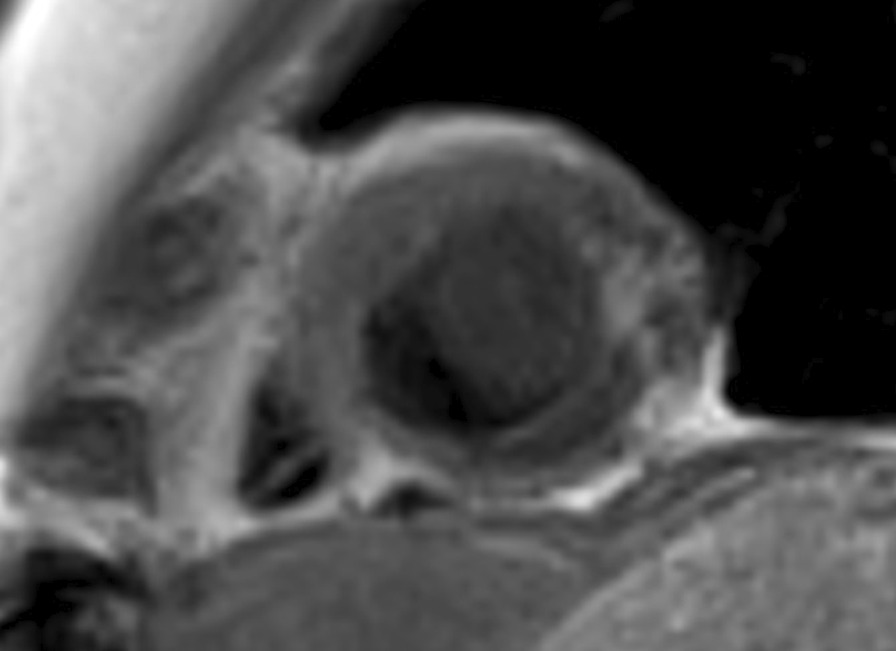
Case 10: Figure 1. Short-axis T2 weighted image. Isointense LV assist device felt plug in the LV cavity

Her CMR revealed normal RV and LV dyskinetic apical segments. The CMR also revealed a felt plug placed at the time of LVAD explantation. The felt plug was visualized as a mass at the LV apex measuring 5.2 cm in length, 1.6 cm in width on the localizer images. Two cm was contained within the LV cavity and 3.1 cm was present outside the LV cavity. The mass was isointense with the myocardium on bSSFP cine images (Additional files 37, 38: Case 10 Movies 1, 2) and on T2 weighted turbo spin sequences (Fig. 30: Case 10 Figure 1).

**Fig. 31:**
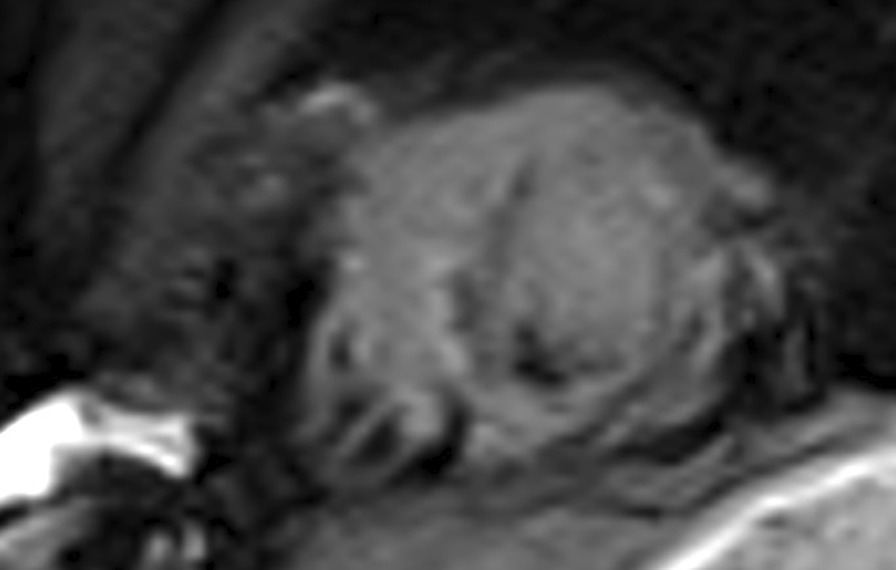
Case 10: Figure 2. Short-axis triple inversion recovery fat suppression. Hyper-intense LV assist device felt plug in the LV cavity

**Fig. 32:**
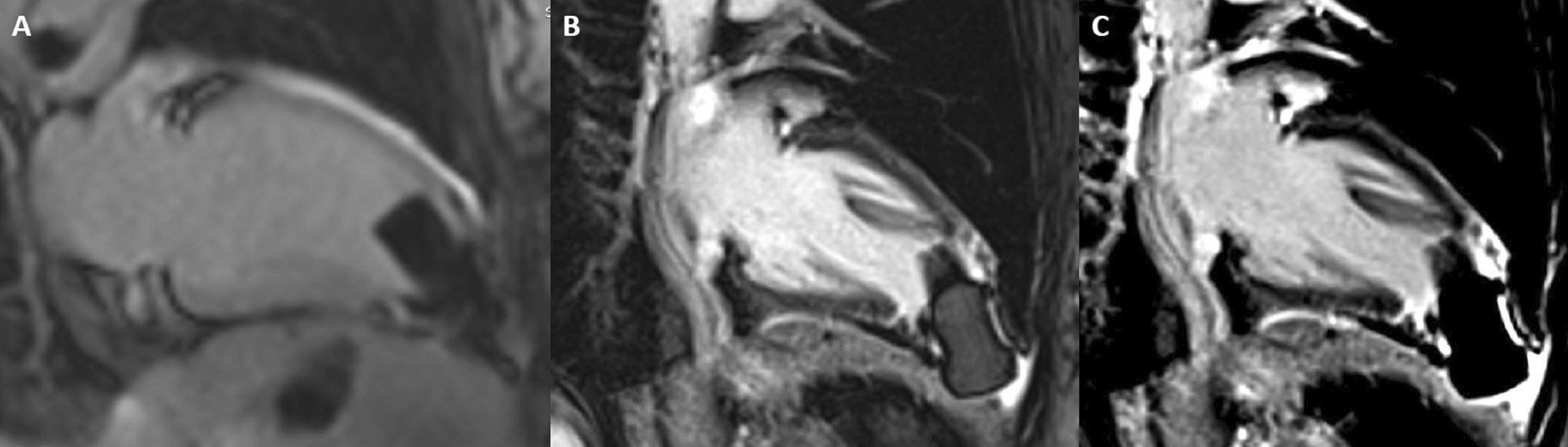
Case 10: Figure 3. LGE with long TI (**A**), myocardial nulling TI magnitude image (**B**), and PSIR (**C**). Hypo-intense on long TI, hyper-intense with an etched appearance on myocardial nulling TI, and hypo-intense on phase sensitive inversion recovery LV assist device (LVAD) felt plug in the LV apex. Transmural LGE of the LV apical wall around the LVAD felt plug

It did not suppress with triple inversion fat suppression sequences (Fig. 31: Case 10 Figure 2) and did not perfuse with contrast on perfusion imaging (Additional file 39: Case 10 Movie 3). On post-contrast high inversion time imaging the plug appears hypointense (Fig. 32: Case 10 Figure 3). On post contrast magnitude LGE imaging with an inversion time to null the myocardium, the felt plug had an etched appearance (Fig. 32: Case 10 Figure 3). On post contrast PSIR sequences, the felt plug was hypointense (Fig. 32: Case 10 Figure 3). Bright scar was present around the felt plug site, due to post-operative changes. A summary of the characteristics of the LVAD felt plug are listed in Table 1: Case 10. As part of the clinical evaluation, the CMR did not reveal any evidence of non-compaction or obvious thrombus.

**Table 1:** Case 10: Magnetic resonance imaging characteristics of the left ventricular assist device felt plug

Sequence	Imaging features
T2 weighted turbo spin echo	Isointense
Post-contrast high inversion time	Hypointense
Post-contrast myocardial nulling inversion time	Hyperintense with an etched appearance
Post-contrast phase sensitive inversion recovery	Hypointense
Cine steady state free precession	Isointense
Perfusion imaging	No perfusion

### Conclusion

**Fig. 33:**
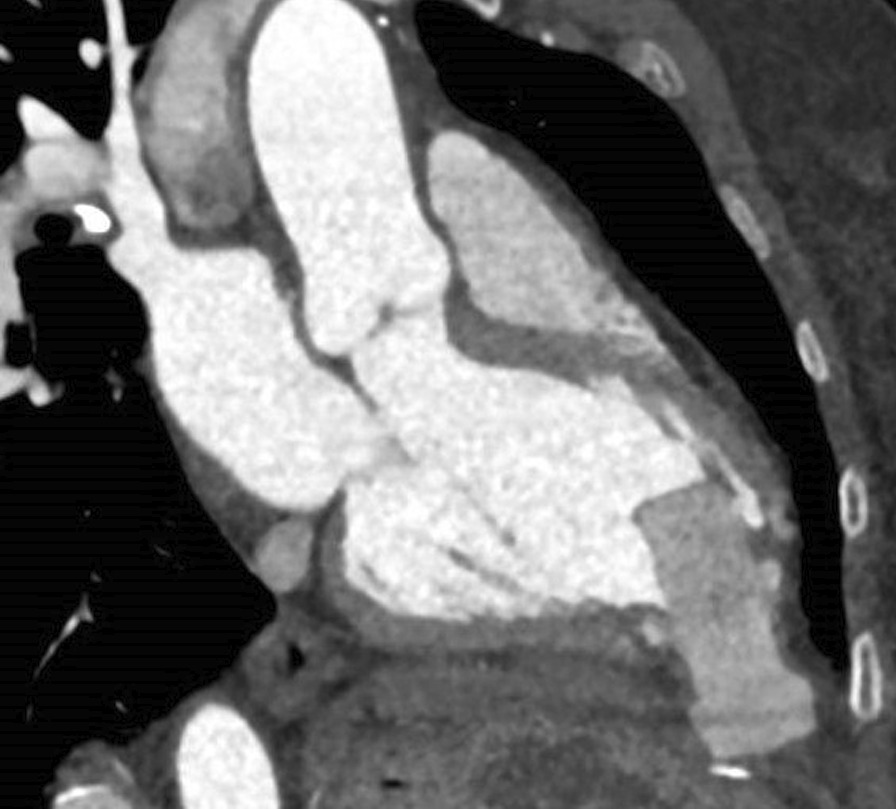
Case 10: Figure 4. Chest CT coronal plane. LV assist device felt plug in the LV apex with no metal present

To the best of our knowledge, this is the first report of CMR imaging of an LVAD felt plug. Although there wasn’t any metal artifact on the CMR, to ensure patient safety for future scans, a CT scan of the chest was performed (Fig. 33: Case 10 Figure 4). With the expanding indications and increasing safety of LVADs, the implantation rates have increased. A small percentage of patients improve their function, leading to removal of the LVAD. Multiple approaches have been described for explantation, including primary closure, patch closure, titanium plug insertion, felt plug insertion and dividing the driveline and leaving the entire pump in situ [66]. CMR studies in patients after LVAD explantation and primary closure reveal apical akinesis and tethering on cine imaging and apical scarring and transmural apical infarction on LGE [67]. This case highlights CMR findings and safety of felt plug imaging with CMR after LVAD explantation.

### Perspective

Although the felt plug was not imaged by CMR in the past, it is CMR safe based on our study. Given the multiple approaches to explantation, it is important for CMR centers to review operative notes, X-rays, and any other imaging available in detail to assess for any metal remnants prior to scanning a patient after LVAD explantation. As there were concern of thrombogenicity of the plug remnants we discussed initiation of anticoagulation with surgeons and heart-failure specialists. Finally, it was decided to not initiate anticogulation. There were no immediate or delayed complications from the procedure. While LVAD presence is an absolute contraindication for CMR, these scans can be safely performed after explantation, even in the case of felt plug placement.

The CMR of Case 10 (Additional file CMR link, https://www.cloudcmr.com/1557-1973-7138-0192/).

## Case 11. CMR evaluation of right atrial mass in an infant with seizure

### Clinical history

**Fig. 34:**
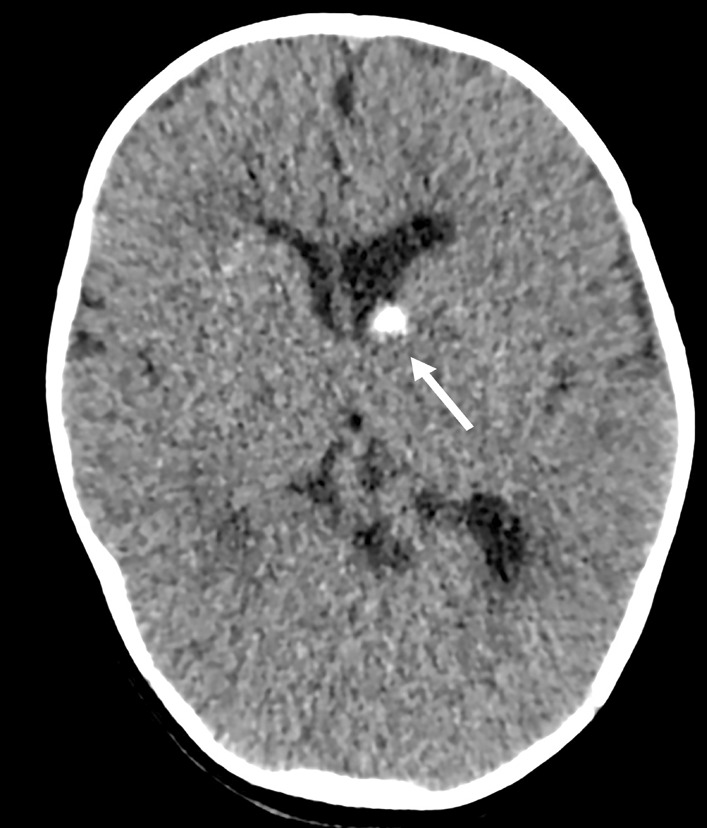
Case 11: Figure 1. Axial brain CT image. Calcified lesion is seen adjacent to left lateral ventricle

**Fig. 35:**
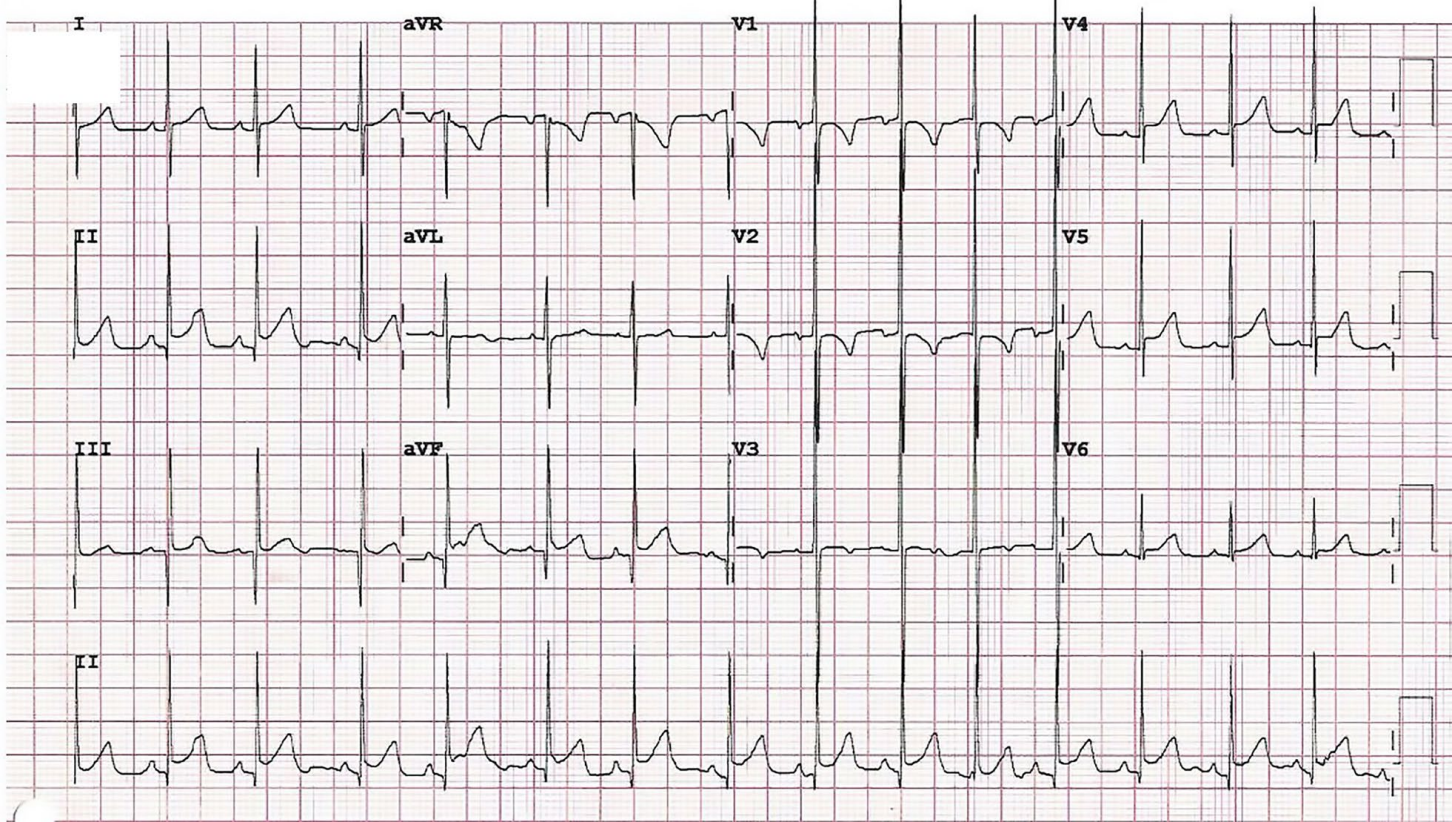
Case 11: Figure 2. Twelve lead ECG demonstrates sinus rhythm with premature atrial contractions

**Fig. 36:**
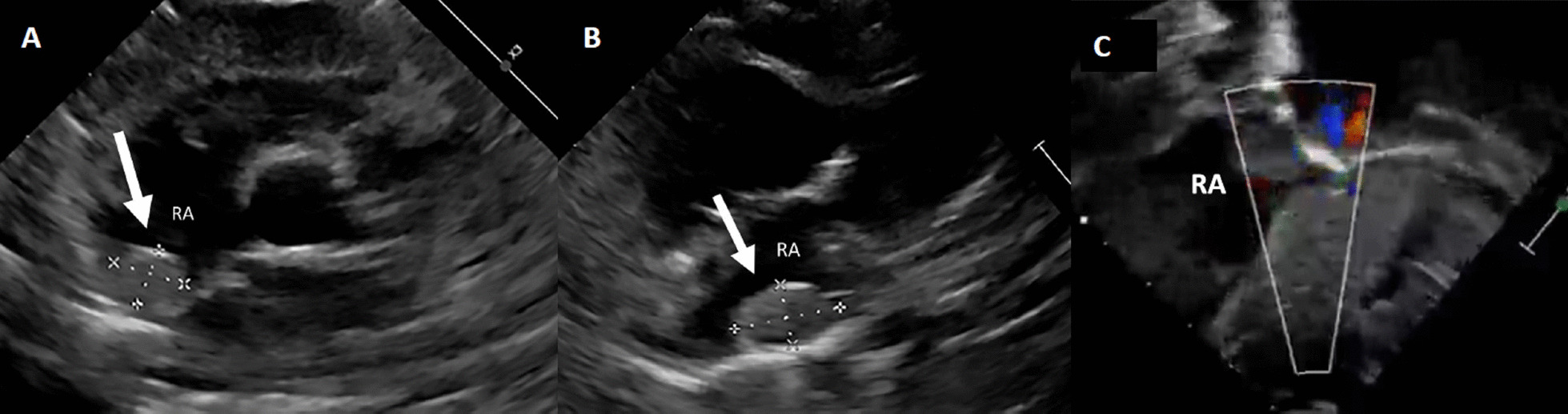
Case 11: Figure 3. TTE parasternal short-axis (**A**), high right parasternal (**B**), and subcostal sagittal (**C**) views. An intracavitary mass (arrow) is noted within the RA between the posterior RA wall and the interatrial septum. The mass is seen at the posterior RA wall, midway between SVC and IVC without systemic venous or tricuspid inflow obstruction. Doppler echocardiography showing lack of vascular flow signal of the RA mass

A 4-month-old girl presented with constipation for 1 month. Clinical history revealed recurrent attacks of flexion spasms. Clinical examination showed generalized hypopigmented macules over the body and trunk. A non-contrast CT brain was performed, showing calcified lesions adjacent to the left lateral ventricle, which were suspicious of calcified subependymal hamartoma (Fig. 34: Case 11 Figure 1). Baseline ECG showed sinus rhythm with premature atrial contraction (Fig. 35: Case 11 Figure 2). TTE was subsequently performed which showed a 1.5 cm × 0.7 cm wide-based oval mass within the RA, between the posterior RA wall and posterior interatrial septum (Fig. 36: Case 11 Figure 3, Additional file 40: Case 11 Movie 1). It was located close to the SVC opening but did not appear to obstruct the tricuspid valve or systemic inflow. A tiny echogenicity was also observed in the LA (images not shown).

### CMR findings

**Fig. 37:**
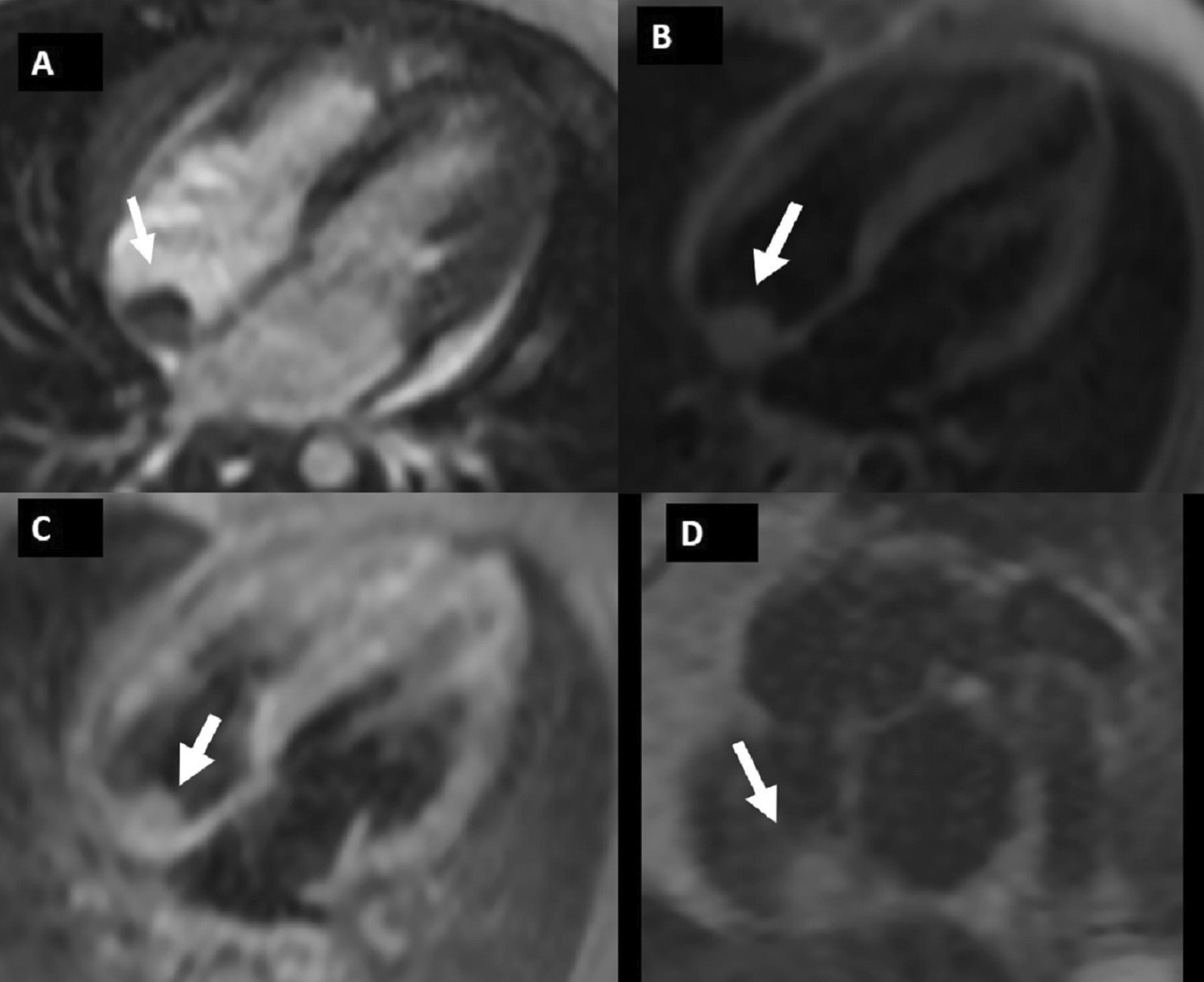
Case 11: Figure 4. T2 bSSFP (**A**) T1 turbo spin echo (**B**) T1 with fat saturation LGE (**C**) four-chamber and T2 triple inversion recovery short axis (**D**). The RA mass (arrow) is isointense on T2 bSSFP, hyper-intense on T1 TSE, hyper-intense on T1 fat saturation LGE, and hyper-intense on T2 TIR. The RA mass abuts the posterior atrial wall and is located close to the interatrial septum. There is no contrast enhancement of the mass

**Fig. 38:**
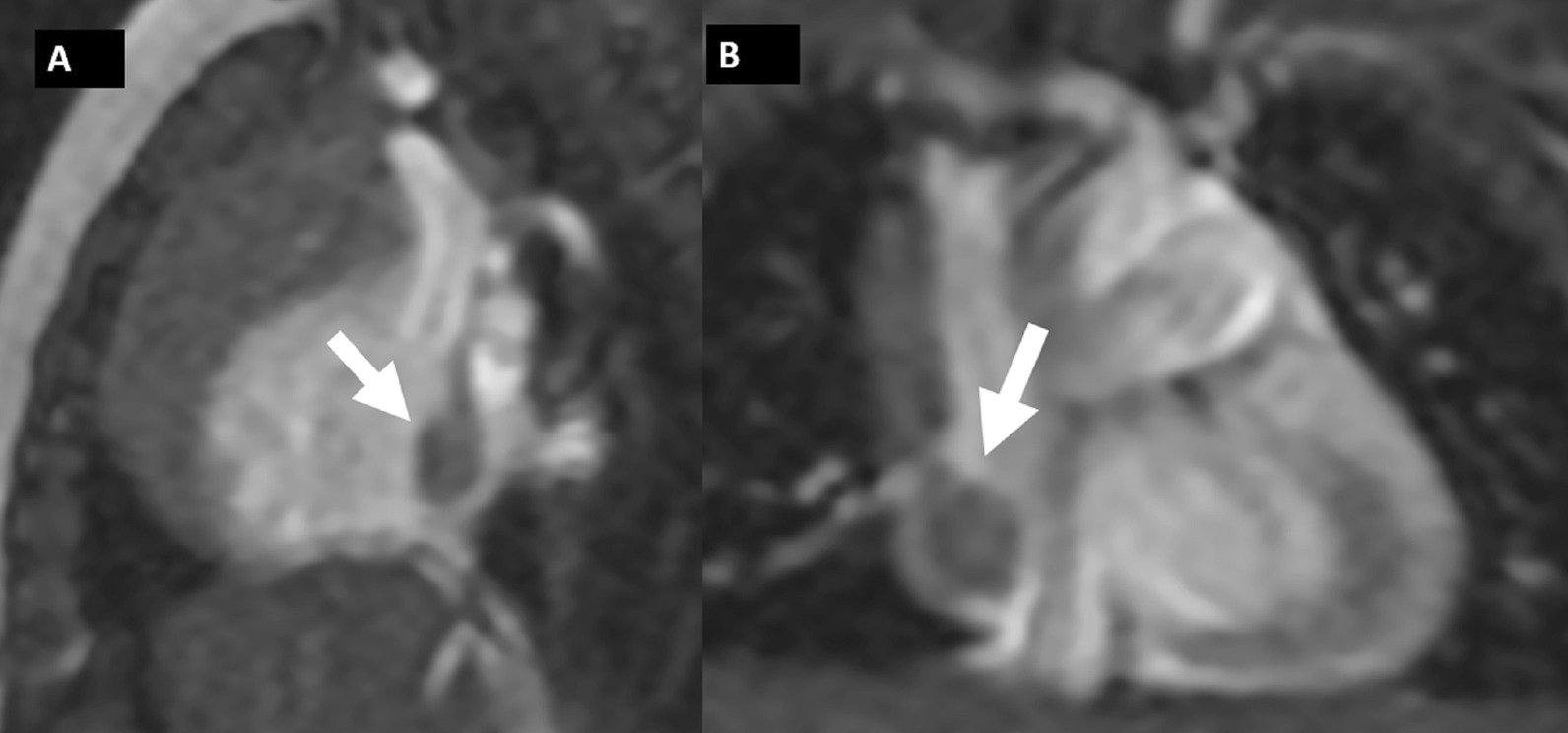
Case 11: Figure 5. T2 steady state free precession sagittal (**A**) and coronal (**B**). The relationship of the mass (arrow) with the superior vena cava and inferior vena cava is shown. There is no obstruction to systemic venous flow

**Fig. 39:**
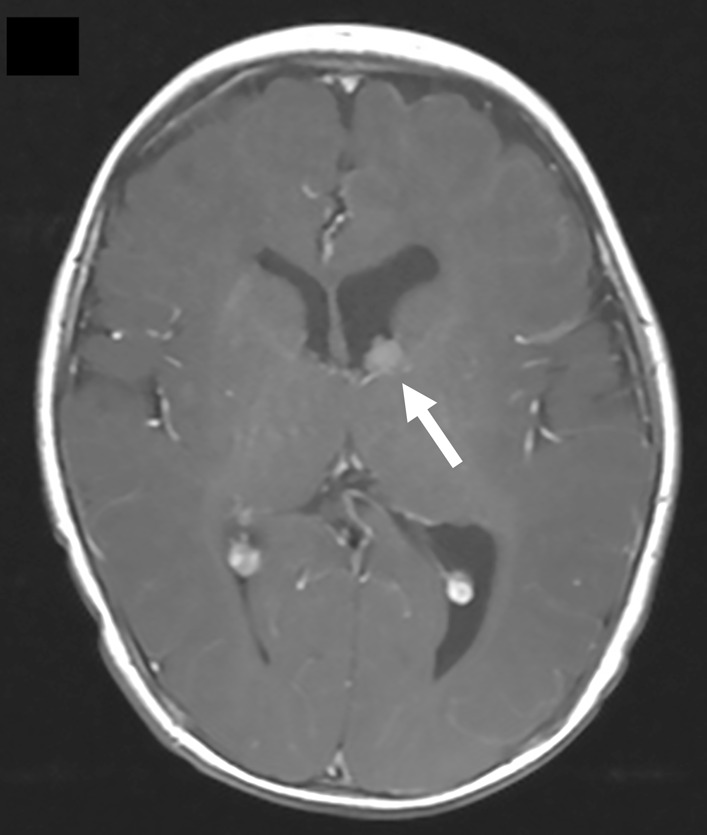
Case 11: Figure 6. Brain MRI T1 SE post gadolinium contrast. Avidly enhancing mass at the anterior left lateral ventricle suspicious of subependymal giant cell astrocytoma (arrow)

CMR and brain MRI were done to evaluate the intracardiac and intracranial lesions, respectively. CMR was mainly used to characterize the cardiac mass and exclude concomitant masses. It showed a solitary RA mass abutting the posterior RA wall and located close to the interatrial septum (Fig. 37: Case 11 Figure 4). It appeared homogenously isointense to myocardium on both T1 weighted and T2 weighted sequences and hyperintense on STIR images. The mass did not enhance on post-contrast images (Fig. 37: Case 11 Figure 4). There was no significant mass effect exerted, as shown in the cine images (Additional files 41, 42: Case 11 Movies 2, 3). Systemic venous inflow from both SVC and IVC were unobstructed (Fig. 38: Case 11 Figure 5). The tricuspid valve was not involved and showed a normal opening. No extracardiac extension was seen. Previously sonographically suspected left atrial lesion was not identified on CMR. Brain MRI performed after CMR that showed multiple small subependymal hamartomata and a larger, avidly enhancing mass highly suspicious of subependymal giant cell astrocytoma (Fig. 39: Case 11 Figure 6).

### Conclusion

Genetic testing subsequently confirmed the diagnosis of tuberous sclerosis. Patient was started on Everolimus due to the presence of intracranial subependymal giant cell astrocytoma. Patient tolerated the medication with good drug compliance.

### Perspective

Cardiac rhabdomyoma is the initial presenting feature of tuberous sclerosis in up to 59% of patients [68]. They are benign hamartomas of the myocardium, 33–50% of which regress within the first 2–4 years of life [69–71]. They are commonly intramyocardial or intracavitary, and located within the ventricles, making the RA location of our case rare [72, 73]. Cardiac rhabdomyoma is usually asymptomatic but may present with outflow tract obstruction, arrhythmia, and heart failure. Multiple masses occur in up to 60% of cases, particularly if associated with tuberous sclerosis [74, 75].

On TTE, rhabdomyoma generally appears as a hyperechoic solid mass. On CMR, they appear isointense to hyperintense on T1 weighted images and hyperintense on T2 weighted images. They are hypo-enhancing on contrast administration relative to the myocardium. TTE and CMR do not entail ionizing radiation, which is particularly important in children. They can be complementary to each other as small tumors (< 0.5 cm) or completely intramural lesions may not be detected by CMR, which is superior in detecting tumors within the cavities and for extracardiac extension assessment, particularly in preoperative cases. CMR can be highly accurate in predicting tumor type in children (Table 2: Case 11) presenting with cardiac mass [72]. In our case, CMR was useful to exclude multiple masses and to confirm and accurately characterize suspicious findings seen on echocardiogram. Additionally, brain MRI was necessary for assessing cerebral mass, which was possible to perform at the same time, allowing optimal pre-treatment planning.

**Table 2:** Case 11: Common pediatric cardiac mass characteristics by cardiac magnetic resonance

	Age	Location	CMR features	Remarks
Benign				
Rhabdomyoma	< 4 years	Intramyocardial or intracavitary; any chamber	Homogeneous on all sequences; no enhancement	Frequent association with tuberous sclerosis
Fibroma	Majority < 1 year	Ventricular septum or free wall	Peripheral enhancement; signal void due to calcifications	Associated with Gorlin syndrome
Teratoma	Children	Pericardial; Usually left atrium if intramyocardial (rare)	Multiloculated mixed solid cystic mass	
Hemangioma	Any age	Ventricular septum or right atrium; intramural or endocardial	T2 hyperintense with heterogeneous enhancement; hyperintense on perfusion sequence	
Malignant				
Lymphoma	Older children and adolescents	Right atrium and right ventricle more common	Lobulated enhancing mass	Typically non-Hodgkin lymphoma
Sarcoma	Young children	Right and left atria more common	Infiltrative irregular mass with heterogeneous enhancement; hemorrhagic pericardial effusion may be present	
Metastasis	Any age	Right heart	Variable features depending on primary tumor	
Non-neoplastic				
Thrombus	Depends on underlying etiology	Commonly left ventricle; Right atrium in the presence of central venous catheter	Variable signal depending on age of thrombus; no enhancement (surface enhancement may be present in chronic organized thrombus)	Commonly associated with indwelling central venous catheters, congenital heart disease and Kawasaki’s disease

CMR should not be routinely performed in all cases of cardiac rhabdomyoma in infants. TTE is often adequate for evaluation of cardiac rhabdomyoma. The consensus statement from the Tuberous Sclerosis Alliance recommends that CMR in infants should be limited by necessity, due to its need for general anesthesia or sedation [76]. The use of CMR should be considered on a case-by-case basis. In our case, the diagnosis of tuberous sclerosis was not yet established at the time of imaging and there was doubt on the TTE diagnosis due to its less common right atrial location and solitary nature. CMR was performed with patient under sedation and we managed to obtain good quality images within a relatively short scanning time (under 30 min).

Treatment is often not necessary as many masses gradually regress. However, surgery may be indicated in patients with severe inflow or outflow obstruction or arrhythmia [77]. Everolimus, a mammalian target of rapamycin (mTOR) inhibitor, has been documented on various case studies to be effective and safe in treating patients with cardiac rhabdomyoma, particularly in tumors which are not resectable [77]. In our patient, the indication for use of Everolimus was primarily for early-onset intracranial subependymal giant cell astrocytoma instead. One would expect the cardiac rhabdomyoma to shrink in our patient as well, both due to its natural course of the disease and the use of Everolimus. TTE can be used for follow-up to document shrinkage or resolution.

CMR is a useful technique in the assessment and follow-up of pediatric cardiac masses. Even though most cardiac masses in tuberous sclerosis are presumed to be rhabdomyoma and spontaneous resolution is often expected, CMR may sometimes be a useful adjunct to TTE in selected cases and is useful in detecting multiple masses, inflow/outflow obstruction, and evaluation for an extracardiac extension.

The CMR of Case 11 (Additional file CMR link, https://www.cloudcmr.com/3857-1973-9938-0189/).

## Supplementary Information

**Additional file 1: Case 1 Movie 1.** Transthoracic echocardiogram (TTE) subcostal coronal sweep and four chamber view. A significant left atrial (LA) to right atrial (RA) shunt at the inferior, posterior septum and a dilated coronary sinus.

**Additional file 2: Case 1 Movie 2.** CMR Axial cine bSSFP. Likely unroofed coronary sinus.

**Additional file 3: Case 1 Movie 3.** Two chamber cine bSSFP. Communication between the enlarged coronary sinus and the LA.

**Additional file 4: Case 1 Movie 4.** 4D flow imaging. Left anterior oblique (top left), transverse (top right), and sagittal (bottom left) views of the atrial level shunt demonstrating flow from the LA into the enlarged coronary sinus and then into the RA through the os of the coronary sinus. A 3D rendering (bottom right) more clearly visualizes the normal pulmonary venous return to the LA and the significant left to right shunt.

**Additional file 5: Case 1 Movie 5.** 4D flow imaging. A 3D rendering in a 4 chamber view showed the substantial flow from the left atrium across the inferior atrial septum into the RA.

**Additional file 6: Case 1 Movie 6.** Intraoperative transesophageal echocardiogram (TEE). A pre-operative, mid-esophageal view of the interatrial septum demonstrating the large coronary sinus atrial septal defect and left to right shunting.

**Additional file 7: Case 1 Movie 7.** Post-operative TEE. Post-operative, mid-esophageal view showing the patch over the os of the coronary sinus. It is placed deeper into the os to avoid damage to the conduction system.

**Additional file 8: Case 2 Movie 1.** Four chamber cine bSSFP. An enlarged RA and ventricle with a secundum atrial septal defect with left to right shunt.

**Additional file 9: Case 2 Movie 2.** Coronal velocity encoded image. A moderate secundum atrial septal (ASD) defect measuring 1.5 x 1.3 cm.

**Additional file 10: Case 2 Movie 3.** Short axis cine bSSFP. The mid-ventricular level illustrating mild to moderate RV dilation with normal systolic function.

**Additional file 11: Case 2 Movie 4.** ECG gated contrast angiogram in a coronal plane. Right sided aortic arch with a left descending aorta and aberrant left subclavian artery.

**Additional file 12: Case 2 Movie 5.** ECG gated contrast angiogram in an axial plane. Right sided aortic arch with a left descending aorta and aberrant left subclavian artery.

**Additional file 13: Case 2 Movie 6.** 3D reconstruction of aortic arch. Right sided aortic arch with a left descending aorta and aberrant left subclavian artery.

**Additional file 14: Case 3 Movie 1.** Transthoracic echocardiography (TTE) apical four-chamber view. RV dilation and diastolic septal shift suggestive of RV volume overload.

**Additional file 15: Case 3 Movie 2.** TTE parasternal short-axis view. RV dilation and diastolic septal shift suggestive of RV volume overload.

**Additional file 16: Case 3 Movie 3.** TTE parasternal short axis with color Doppler. Pulmonic regurgitation is present but difficult to quantify.

**Additional file 17: Case 3 Movie 4.** CMR cine bSSFP short axis view. Basal septal thinning, systolic septal dyskinesis, and diastolic septal flattening.

**Additional file 18: Case 3 Movie 5.** CMR cine bSSFP RV long-axis view. Global RV systolic dysfunction and moderate pulmonic regurgitation.

**Additional file 19: Case 3 Movie 6.** CMR cine bSSFP 4 chamber view. RA and RV dilation, RV systolic dysfunction, basal septal systolic dyskinesis, and diastolic septal flattening.

**Additional file 20: Case 4 Movie 1.** TTE four chamber view. RV dilation with normal bi-ventricular systolic function.

**Additional file 21: Case 4 Movie 2.** Time resolved 3D MRA in the coronal plane. A persistent left sided SVC is readily identified and seen draining into the coronary sinus, which drains into the RA. The azygous continuation of the interrupted inferior vena cava is well seen. The azygos continuation of IVC coursing along the right side of the descending aorta and then draining into the right SVC at the right paratracheal space. The hemiazygous vein is seen draining into the persistent left sided vena cava.

**Additional file 22: Case 4 Movie 3.** Time resolved 3D MRA in the axial plane. A persistent left sided SVC is readily identified and seen draining into the coronary sinus, which drains into the RA. The azygous continuation of the interrupted inferior vena cava is well seen. The azygos continuation of IVC coursing along the right side of the descending aorta and then draining into the right SVC at the right paratracheal space. The hemiazygous vein is seen draining into the persistent left sided vena cava.

**Additional file 23: Case 4 Movie 4.** Time resolved 3D MRA in the sagittal plane. A persistent left sided SVC is readily identified and seen draining into the coronary sinus, which drains into the RA. The azygous continuation of the interrupted IVC is well seen. The azygos continuation of IVC coursing along the right side of the descending aorta and then draining into the right SVC at the right paratracheal space. The hemiazygous vein is seen draining into the persistent left sided vena cava.

**Additional file 24: Case 5 Movie 1.** Multi-echo GRE fat water separation sequence out of phase cine.

**Additional file 25: Case 5 Movie 2.** Multi-echo GRE fat water separation sequence fat only cine.

**Additional file 26: Case 5 Movie 3.** Cine SSFP four chamber. Hypointense regions within the subendocardium corresponding to scattered areas of fat deposition.

**Additional file 27: Case 5 Movie 4.** First pass perfusion 4 chamber. Subendocardial perfusion abnormality which corresponds to areas of LGE.

**Additional file 28: Case 5 Movie 5.** First pass perfusion 2 chamber. Subendocardial perfusion abnormality which corresponds to areas of LGE.

**Additional file 29: Case 6 Movie 1.** Cine bSSFP four chamber. Isointense mass on the tricuspid valve.

**Additional file 30: Case 6 Movie 2.** Cine bSSFP short axis. Isointense mass on the tricuspid valve.

**Additional file 31: Case 7 Movie 1.** Cine SSFP sagittal. Subvalvar RV outflow tract narrowing in the setting of a pectus deformity.

**Additional file 32: Case 7 Movie 2.** In-plane phase contrast right ventricular outflow tract. Evidence of subvalvar RV outflow tract obstruction.

**Additional file 33: Case 7 Movie 3.** Cine bSSFP axial. Significant subvalvar narrowing of the RV outflow tract.

**Additional file 34: Case 8 Movie 1.** Cine bSSFP four chamber. Mitral valve prolapse (MVP), mitral annular disjunction (MAD), and moderate mitral regurgitation.

**Additional file 35: Case 8 Movie 2.** Cine bSSFP three chamber. MVP, mitral annular disjunction (MAD), and moderate mitral regurgitation.

**Additional file 36: Case 8 Movie 3.** Cine SSFP two chamber. MVP, MAD, and moderate mitral regurgitation.

**Additional file 37: Case 10 Movie 1.** Cine bSSFP two chamber. Isointense LV assist device felt plug in the LV apex. Normal LV systolic function with apical akinesia.

**Additional file 38: Case 10 Movie 2.** Cine bSSFP three chamber. Isointense LV assist device felt plug in the LV apex. Normal LV systolic function with apical akinesia.

**Additional file 39: Case 10 Movie 3.** First pass perfusion four chamber. No contrast uptake of the LV assist device felt plug in the LV apex.

**Additional file 40: Case 11 Movie 1.** TTE parasternal short axis. Mass in the RA.

**Additional file 41: Case 11 Movie 2.** Cine bSSFP four chamber. The mass in the RA is isointense.

**Additional file 42: Case 11 Movie 3.** Cine bSSFP short axis. The mass in the RA is isointense.

## Data Availability

All data generated or analyzed during this study are included in this published article [and its additional information files].

## Abbreviations

AAo: Ascending aorta
ACS: Acute coronary syndrome
AP: Anteroposterior
ARVC: Arrhythmogenic right ventricular cardiomyopathy
AS: Aortic stenosis
ASD: Atrial septal defect
bSSFP: Balanced steady state free precession
CAD: Coronary artery disease
CHD: Congenital heart disease
CMR: Cardiovascular magnetic resonance
CT: Computed tomography
DAo: Descending thoracic aorta
ECG: Electrocardiogram
EFE: Endocardial fibroelastosis
EGE: Early gadolinium enhancement
EGPA: Eosinophilic granulomatosis with polyangiitis
FIDDLE: Flow-independent dark-blood delayed enhancement
GRE: Gradient echo
HCM: Hypertrophic cardiomyopathy
ICD: Implantable cardioverter defibrillator
IVC: Inferior vena cava
LA: Left atrium/left atrial
LE: Loeffler’s endocarditis
LGE: Late gadolinium enhancement
LSVC: Left superior vena cava
LV: Left ventricle/left ventricular
LVAD: Left ventricular assist device
LVEF: Left ventricular ejection fraction
MAD: Mitral annular disjunction
MI: Myocardial infarction
MINOCA: Myocardial infarction with non-obstructive coronary arteries
MRA: Magnetic resonance angiography
MRI: Magnetic resonance imaging
mTOR: Mammalian target of rapamycin
MV: Mitral valve
MVO: Microvascular obstruction
MVP: Mitral valve prolapse
PC: Phase contrast
PCI: Percutaneous coronary intervention
PDGF: Platelet derived growth factor
PR: Pulmonic regurgitation
PS: Pulmonic stenosis
PSIR: Phase sensitive inversion recovery
RA: Right atrium/right atrial
RV: Right ventricle/right ventricular
RVEDVI: Right ventricular end-diastolic volume index
RVEF: Right ventricular ejection fraction
SCMR: Society for Cardiovascular Magnetic Resonance
SGCA: Subependymal giant cell astrocytoma
STIR: Short tau inversion recovery
SVC: Superior vena cava
TAO: Thoracic aortic arch
TEE: Transesophageal echocardiogram
TI: Inversion time
TTE: Transthoracic echocardiogram
VSD: Ventricular septal defect
VT: Ventricular tachycardia
